# Simultaneous delimitation of species and quantification of interspecific hybridization in Amazonian peacock cichlids (genus *cichla*) using multi-locus data

**DOI:** 10.1186/1471-2148-12-96

**Published:** 2012-06-22

**Authors:** Stuart C Willis, Jason Macrander, Izeni P Farias, Guillermo Ortí

**Affiliations:** 1 School of Biological Sciences, 348 Manter Hall, University of Nebraska-Lincoln, Lincoln, NE, 68588, USA; 2 Department of Evolution, Ecology, and Organismal Biology, The Ohio State University, 318 W. 12th Avenue, Columbus, OH, 43210, USA; 3 Laboratório de Evolução e Genética Animal, ICB, Universidade Federal do Amazonas, Estrada do Contorno 3000, Manaus, AM, Brazil; 4 Department of Biology, The George Washington University, 2023G St. NW Suite 340, Washington, DC, 20052, USA

## Abstract

**Background:**

Introgression likely plays a significant role in evolution, but understanding the extent and consequences of this process requires a clear identification of species boundaries in each focal group. The delimitation of species, however, is a contentious endeavor. This is true not only because of the inadequacy of current tools to identify species lineages, but also because of the inherent ambiguity between natural populations and species paradigms. The result has been a debate about the supremacy of various species concepts and criteria. Here, we utilized multiple separate sources of molecular data, mtDNA, nuclear sequences, and microsatellites, to delimit species under a polytypic species concept (PTSC) and estimate the frequency and genomic extent of introgression in a Neotropical genus of cichlid fishes (*Cichla*). We compared our inferences of species boundaries and introgression under this paradigm to those when species are identified under a diagnostic species concept (DSC).

**Results:**

We find that, based on extensive molecular data and an inclusive species concept, 8 separate biological entities should be recognized rather than the 15 described species of *Cichla*. Under the PTSC, fewer individuals are expected to exhibit hybrid ancestry than under the DSC (~2% vs. ~12%), but a similar number of the species exhibit introgression from at least one other species (75% vs. 60%). Under either species concept, the phylogenetic breadth of introgression in this group is notable, with both sister species and species from different major mtDNA clades exhibiting introgression.

**Conclusions:**

Introgression was observed to be a widespread phenomenon for delimited species in this group. While several instances of introgressive hybridization were observed in anthropogenically altered habitats, most were found in undisturbed natural habitats, suggesting that introgression is a natural but ephemeral part of the evolution of many tropical species. Nevertheless, even transient introgression may facilitate an increase in genetic diversity or transfer of adaptive mutations that have important consequences in the evolution of tropical biodiversity.

## Background

Throughout the New Synthesis, hybridization in animals was relegated to a minimal role in evolutionary theory e.g. [1], often only considered important for reinforcing reproductive isolation through the reduced fitness of hybrid offspring [2]. More recently, it has been recognized that hybridization is actually quite common, with 6-10% of animal species engaging in heterospecific mating [3], and the introgressive consequences of this hybridization are frequently encountered in surveys of genetic diversity [4]. Introgressive hybridization, or the movement of DNA from one species to the gene pool of another species by repeated backcrossing of hybrid individuals with one or both parent species, could be an important source of novel variation for a population that is thus less constrained by in situ mutation [5]. However, it remains unclear how often hybridization occurs within an individual group of closely related species, both in terms of the number of species that hybridize and the proportion of individuals with hybrid ancestry. Further, while it has been observed that heterospecific mitochondria may commonly invade the gene pool and even replace the native mtDNA of a species following hybridization [4,6,7], the proportion of the nuclear genome that is affected by introgression has rarely been documented e.g. [8]. Addressing these issues is critical for a comprehensive evolutionary theory, particularly since the consequences of hybridization may go well beyond reinforcement to include adaptive introgression, adaptive radiation, hybrid speciation, and fusion or extinction of poorly isolated species [3,5,9].

Estimating introgressive hybridization requires a clear understanding of species boundaries, a requirement often vulnerable to the continued controversy over the species paradigm [10,11]. Species are generally considered to be groups of interbreeding individuals (populations) that exchange genetic material with minimal functional constraint (e.g. phenotypes that after recombination that are inviable or sterile) and more exclusively with con-specifics than with other groups of individuals, and as a result, show more phenotypic (morphological and functional) similarity and experience a constrained group-wise evolutionary trajectory sensu reviews by [12,13]. However, within this paradigm there is considerable debate about the relative merit of criteria used for the identification of groups of individuals classified as species taxa or the products of speciation. The two most commonly referenced species concepts, the biological e.g. [14] and phylogenetic (or diagnostic, hereafter DSC) [15] species concepts, emphasize the efficacy of intrinsic isolating barriers versus the presence of autapomorphic characters distinguishing populations, respectively, and in practice will often identify conflicting sets of species taxa [11,16]. As a result, it remains unclear what type of reproductive pairings and gene flow qualify as introgressive hybridization, that is, when gene exchange is interspecific rather than intraspecific. Nevertheless, despite the ambiguous correspondence between species concepts and natural groups, most biologists implicitly or explicitly consider species to be real entities reflecting the discontinuous nature of biological variation [17], and individuals ascribed to a given species are often treated interchangeably in an array of biological investigations [18-21]. This implies an expectation that there should be some natural distinction between introgressive hybridization and intraspecific gene flow, a conjecture that can be investigated in nature by surveying large numbers of individuals and examining them for phenotypic and genetic disjunctions [i.e. multimodality along continuous axes of variation; 10]. Ideally, it should be directly clear from this kind of data what qualifies as introgression rather than intraspecific gene exchange [22], but it is also possible to consider inferences about hybridization and introgression in light of different species concepts and criteria [23].

Here, we investigate species boundaries and the frequency and extent of hybridization in a genus of Neotropical cichlid fishes using multi-locus data under two alternative species concepts. The Neotropical region is home to the largest assemblage of freshwater fishes, representing the richest assemblage of vertebrates on earth (~10% of vertebrate diversity) [24]. Our analysis focused on the genus *Cichla* Schneider, 1801, commonly known as peacock basses. There are 15 recognized species of *Cichla *[25], all of equal karyotype, 2 N = 48 [26] and references therein. These fishes are large bodied (up to 12 Kg), diurnal piscivores and have repeatedly been implicated in key trophic structuring and nutrient cycling processes in Neotropical floodplain ecosystems [27-29]. Tagging studies in the native distribution of these fishes have shown that most individuals are highly site-fidelous, even across years, but do exhibit occasional long-distance dispersal [30]. *Cichla* are also seasonally monogamous and show extensive parental care [29]. Our null hypothesis for delimitable species units in this genus was given by the 15 species recognized by Kullander and Ferreira [25] using morphological data and a DSC. A study of the mtDNA of these species found a maximum of approximately 7% sequence divergence at the cytochrome *b* gene among these species, which, based on a rough 1% per million years calibration for fishes [31], would suggest initial divergences in the upper Miocene [32]. Unpublished studies of Neotropical cichlid diversification, however, place these divergences approximately 16 Ma ago, or older (H. Lopez-Fernandez, personal comment to SCW). This study also found that more closely related species tended to be allo- or parapatric (adjacent), but sympatry is common among more distantly related species that carry distinct mtDNA lineages (see approximate species distributions in Additional file 1: Figure S1a-d). Most of these sympatric species also exhibit preference for different habitats (allotopy) and other resource partitioning [33], with notable exceptions. Because body shape has a high degree of conservation in this genus, morphological species discrimination by Kullander and Ferreira was often based on subtle color pattern differences, modal differences in meristic variation, and geography [25] (see also Methods). Subsequent work in *Cichla* using mitochondrial DNA showed both congruence and incongruence with morphological estimates of species [34]. In addition, studies examining morphological-mitochondrial mismatch [34], as well as karyology [26,35], have inferred hybridization in natural and in artificial or disturbed environments. Thus, for comparison to the DSC employed by Kullander & Ferreira [25], we also estimated the amount of hybridization using a more inclusive, polytypic species concept (hereafter PTSC, sensu [16] and earlier works cited therein). As utilized here, we recognized species units as groups of individuals that have shared a history of largely exclusive ancestry and group-wise evolutionary dynamics as demonstrated by congruence between exclusive lineages of alleles, genetic clustering in space, and a consistently discriminable morphotype (or other phenotypic traits, e.g. their niche). In practice, this makes the PTSC much like several other species concepts, including the evolutionary species concept [36] and genotypic cluster concept [37]. In fact, it should not be considered a separate concept from the general lineage concept of species [13,38]. However, our use of the term PTSC emphasizes our expectation that species are made up of one or perhaps many sub-populations that may exchange genes, homogenize, and be extirpated through time without significant changes in phenotypic diversity (i.e. meta-populations). Thus, here we considered partially distinct sub-populations, those that are distinct in a subset of the data, still exchange genes, and intergrade at their borders, to be subspecies or evolutionary significant units of more inclusive species units.

In this study, we analyzed and compared evidence provided by mtDNA and nuclear gene genealogies with extensive microsatellite genotyping for a dense taxonomic sample representing all nominal species in the genus. Our purpose was less focused on delimiting species (although we do make some recommendations) than on observing the distribution and congruence of clusters of individuals in multivariate space [sensu [10,39], and discerning how changes in the way we view species affects our interpretation of the rates and significance of introgressive hybridization. We find that despite the differences in the concepts employed for species delimitation, some inferences about introgression appear robust, suggesting that hybridization as a process should not be discounted either in the delimitation of species or studies of adaptation. In addition, based on clear conflicts between published taxonomy and results from analysis of molecular data from many individuals, we recommend that Neotropical fish systematists adopt a strategy where apparent morphological or molecular disjunctions are iteratively and adaptively tested before erecting novel specific categories.

## Methods

### Sampling and species identification

**Figure 1:**
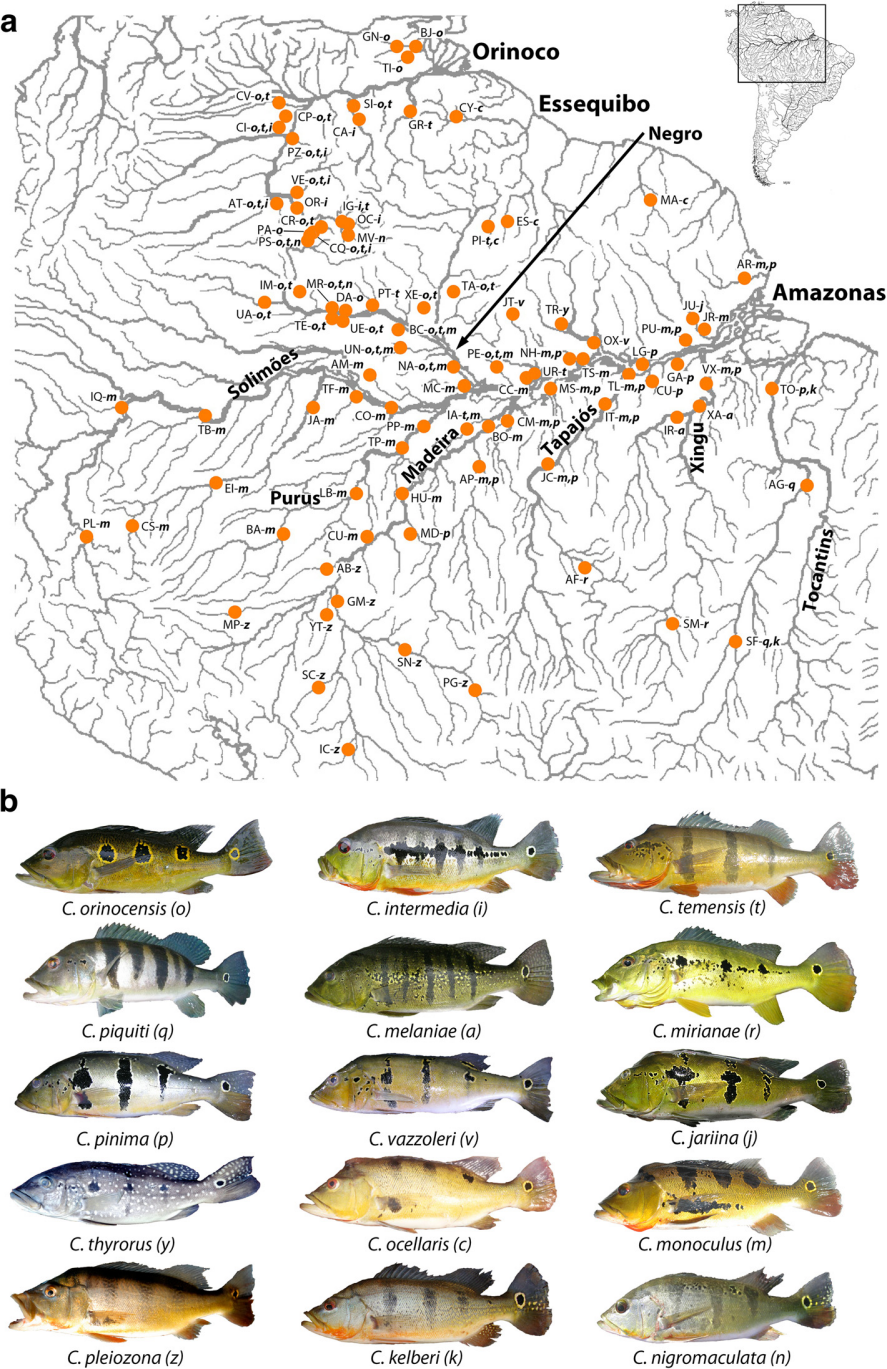
**Sampling Localities and Species Morphologies. a**) Map of sampling localities, with species collected in those localities. Codes for localities and species follow Table 1 and part b of this figure, respectively. **b**) Images showing representative color morphology for the 15 described species of *Cichla.*

We collected fin, gill, or white muscle tissues from fishes in the Amazonas, Orinoco, Essequibo, and Maroni River drainages and preserved them in 90% ethanol or DMSO-EDTA saturated with NaCl (Table 1, Figure 1, Additional file 2: Table S1). Collection permit numbers are listed in the Acknowledgements. We identified each individual according to Kullander and Ferreira [25] using morphology, as possible. Identification was based on the presence, absence, or placement of bars or spots on the head and flank, counts of lateral line scales or proximal scale rows, and body background coloration (Figure 1). Identification of fishes to morphological subgroup (*C. temensis* and remaining clade A species, *C. orinocensisC. intermedia*, or *C. ocellaris* and remaining clade B1 species), which are sympatric, was unambiguous at every locality. Discrimination between species within these groups (clades A and B1) was ambiguous for the allopatric species *C. pinimaC. vazzoleriC. thyrorus*, and *C. jariina*, and similarly for *C. ocellarisC. monoculusC. nigromaculata*, and *C. pleiozona* (the spotting of anal, pelvic, and caudal fins turned out to be adequate to discriminate *C. kelberi*). Our identification of these therefore followed Kullander’s & Ferreira’s description using estimated geographic distributions. Note that we did not reevaluate species boundaries using morphology, but accepted these authors’ conclusions about specific units as our null hypothesis directly.

**Table 1:** Sampling localities and sample sizes for mtDNA/nuclear sequences/microsatellites

	**locality**	** *temensis* *(t)* **	** *pinima* *(p)* **	** *vazzoleri* *(v)* **	** *thyrorus* (y)**	** *jariina* *(j)* **	** *piquiti* *(q)* **	** *melaniae* *(a)* **	** *mirianae* *(r)* **	** *intermedia* *(j)* **	** *orinocensis* *(o)* **	** *ocellaris* *(c)* **	** *monoculus* *(m)* **	** *nigromaculata* *(n)* **	** *kelberi* *(k)* **	** *pleiozona* *(z)* **
TI	Tigre										8/3/10					
GU	Guanipa										4/2/4					
BJ	Buja										2/-/2					
GR	Guri Reservoir (Caroni)	11/1/8														
SI	Sipao	10/-/10									10/5/10					
CA	Caura									15/1/12						
CV	Cunavichito	1/-/1									3/-/3					
CP	Capanaparo	10/-/10									10/2/10					
CI	Cinaruco	12/1/26								10/1/10	11/3/11					
PZ	Parguaza	2/-/2								2/2/2	12/-/12					
AT	Atabapo	10/1/10								2/-/2	10/1/10					
VE	Ventuari	9/-/9								12/1/12	12/2/25					
OR	Orinoco									4/-/4						
IG	Iguapo	1/1/-								10/-/9						
OC	Ocamo									10/1/9						
MV	Mavaca													10/2/10		
CR	Curamoni	3/-/3									7/-/7					
PA	Perro de Agua										16/-/16					
CQ	Casiquiare	1/-/1								15/1/15	2/-/2					
PS	Pasiba	10/1/10									17/4/17			17/4/17		
UA	Uaupes	11/-/10									20/-/20					
IM	Ia-mirim	1/-/-									5/-/5					
TE	Teá	1/-/1									10/-/10			1/-/-		
MR	Marauiá	9/-/7									10/-/10			11/1/10		
UE	Uneiuxi	10/-/5									23/4/10					
DA	Daraá										2/-/2					
PT	Preto	1/-/1														
BC	Barcelos	7/-/7									1/-/1		10/4/10			
PI	Pirara (Takutu)	5/1/5										11/1/11				
ES	Essequibo (Rupununi)											13/3/10				
CY	Cuyuni											1/1/1				
MA	Maroni											2/2/2				
XE	Xeruini	9/2/9									10/6/10					
TA	Tapera	12/2/10									10/5/10					
UN	Unini	16/3/10									12/4/12		12/3/10			
NA	Novo Airão	11/-/9									10/-/10		11/-/10			
PE	Preta da Eva	4/-/4									6/-/6		2/-/2			
UR	Urubu	3/-/3														
PL	Pucallpa												2/-/-			
IQ	Iquitos												8/2/4			
TB	Tabatinga												22/-/9			
JA	Juruá (Carauari)												8/-/-			
EI	Eirunepé												3/-/3			
CS	Cruzeiro do Sul												10/-/10			
AM	Lago Amaná												10/-/9			
TF	Tefé												8/-/7			
CO	Coari												6/-/6			
PP	Piagaçu-Purus												10/-/10			
TP	Tapauá												11/-/10			
LB	Labrea												2/-/2			
BA	Boca do Acre												20/-/10			
MC	Manacapuru												10/-/10			
IA	Igapu-Açu	10/2/10											10/1/10			
BO	Borba												10/-/10			
CM	Canumã		10/1/10										13/-/13			
AP	Aripuanã		13/2/10										3/-/3			
HU	Humaita												9/-/9			
MD	Machado		2/1/2													
CN	Cunia												20/-/10			
CC	Canaçari												10/-/8			
MS	Maués		9/1/8										10/-/7			
JT	Jatapu (Uatumã)			5/5/5												
NH	Nhamunda		10/-/10										10/-/9			
TS	Terra Santa												10/-/10			
TR	Trombetas (abv. rapids)				2/2/2											
OX	Oriximiná			15/2/14												
LG	Lago Grande		9/-/9													
TL	Tapajós mouth		10/2/10													
IT	Itaituba		10/3/9										14/-/9			
JC	Jacareacanga		8/-/8										5/-/5			
CU	Curuá-Una		5/1/5													
PU	Paru		6/1/6										13/-/10			
GA	Guajara		10/-/9													
VX	Vitória do Xingu		4/1/4										10/-/10			
JR	Jari (lower)												10/1/10			
JU	Jari (above waterfalls)					9/5/9										
AR	Araguari		6/1/6										2/1/2			
AF	Alta Floresta								5/5/5							
SM	Suia Missu								10/2/10							
XA	Xingu (Altamira)							2/2/2								
IR	Iriri							19/5/13								
TO	Tocantins (Baião)		4/-/4												10/-/10	
AG	Araguatins						10/-/7									
SF	São Felix do Araguaia						10/5/10								10/5/10	
AB	Abunã															7/-/7
GM	Guajará-Mirim															10/4/9
MP	Manuripi															12/-/-
YT	Yata															9/-/-
SC	Secure															2/-/-
SM	San Martin															6/-/-
IC	Ichilo															4/-/-
PG	Paragua															8/-/-
Totals		190/15/181	116/14/110	20/7/19	2/2/2	9/5/9	20/5/17	21/7/15	15/7/15	80/7/75	243/41/245	27/7/24	324/12/257	32/4/29	20/5/20	58/4/16

Codes for localities and species names are used in Figure 1.

While many vouchers were taken (Additional file 3: Table S2), where possible fishes were photographed, sampled non-destructively (dorsal fin clip), and released alive. Sampling was done between 2003 and 2010. We targeted to collect 10 individuals per locality where possible. DNA was extracted from samples using the Qiagen DNeasy extraction kit (Qiagen Inc.), following manufacturer’s recommendations, and used with a panel of molecular markers to estimate co-ancestry between individuals, and gene flow between localities. Patterns of gene flow are a logical criterion for understanding species limits, but this is not without its limitations. For instance, genetic disjunctions between localities resulting from a spatial genetic structure within a species (e.g. isolation by distance) may be misinterpreted as separate meta-populations (i.e. species) if intervening localities are not sampled. It was therefore important to sample sufficiently densely on an organism-specific scale to observe the connectivity between sub-populations [40]. Further, we found that it was impossible to determine an adequate sampling strategy entirely *a priori*. Rather, we found it was necessary to sample repeatedly with a focus on testing genetic discontinuities discovered in preliminary analyses of the data.

### Molecular markers

We collected data from three different sources. First, for every sample we sequenced the mitochondrial control region (mtCR), and for many samples chosen to maximize geographic distribution across species and to confirm mtDNA introgression, we also sequenced the mitochondrial ATPase 8,6 gene (mtATP). The mtCR, with one of the fastest mutation rate in the genome, provides an unambiguous assessment of genealogical connection between individuals in order to estimate intraspecific gene exchange or introgression [40]. The mtATP, on the other hand, shows a more restricted rate of variation due to its utility as a protein-coding gene for the energetic cellular machinery, and provides a way to mitigate effects of homoplasy or alignment-ambiguity among more dissimilar haplotypes in the mtCR dataset [40]. While we could have sequenced every individual for the mtATP, the strict linkage and reduced variation of this gene did not warrant the additional resources, since no significant information advance was likely to be gained [40]. Second, in order to further test patterns of gene flow, we also obtained nuclear DNA sequence data. We sequenced two nuclear loci (each gene fragment targeted was ~750 bp) consisting of both open reading frame and intron segments: a tyrosine kinase gene (Xsrc) [41] and the micropthalamia b receptor protein (Mitf) [42]. Due to the restricted degree of variation, we sequenced these loci from a subset of all individuals, again chosen to maximize geographic distribution across species. While an appropriate complement to mtDNA, the longer coalescence time of nuclear loci means that it can be difficult to distinguish gene exchange from the sorting of ancestral polymorphism among recently diverged species [43-45]. Finally, we genotyped most individuals for a panel of 12 microsatellite loci. This source of nuclear data hypothetically suffers the same constraint as the sequenced nuclear loci: a slow rate of coalescence. However, while lineage sorting of any nuclear locus may be a slow process, deviations from Hardy-Weinberg and linkage equilibrium among alleles at these hypervariable loci can occur among isolated populations over many fewer generations [46]. While each of these datasets has weaknesses and limitations, we used them in combination to make a more accurate estimate of inter-locality gene exchange and species boundaries, expecting that their collective strengths would help counter their individual weaknesses.

We collected data from the mtCR (~550 bp) and mtATP (842 bp) using previously described primers and conditions [32,34]. Many of these data were published previously on Genbank (CR: DQ841819-DQ841946, GU295709–GU295740; ATP: GU295741-GU295801). In addition, we obtained the sequence data generated by another study of *Cichla*[47] in Bolivia and Peru (DQ778661-DQ778712). Sequences were checked and assembled using CodonCode Aligner (CodonCode Corp.) and MacClade (Maddison & Maddison 2000).

PCR reactions for Xsrc and Mitf contained 20 mM Tris–HCl (pH 8.4), 50 mM KCl, 2.0 mM MgCl_2_, 200 μM each dNTP, 0.1 μM each primer, 1.5 μL of 20 mg/mL bovine serum albumin (Fermentas), 0.5 U of Takara ExTaq polymerase (with proof-reading exonuclease activity), and 3 to 4 μL DNA extract (10–50 ng⁄μL) in 30 μL reaction volumes. Published primers were used. Thermal cycling conditions for Mitf on an MJ Research PTC200 thermal cycler were 1 min at 94°C, 35 cycles of 30 sec at 94°C, 30 sec at 54°C, and 1.5 min at 72°C, followed by 10 min at 72°C. Thermal cycling of Xsrc required a touchdown protocol of 1 min at 95°C, 30 cycles of 15 sec at 98°C, 30 sec at *X*°C, 1.5 min at 72°C, followed by 10 min at 72°C, where *X* was 64°C for 3 cycles, 62°C for 3 cycles, 60°C for 3 cycles, 58°C for 6 cycles and 52°C for 15 cycles. PCR products were sequenced at the University of Washington High Throughput Facility. Chromatograms were checked and assembled using CodonCode Aligner. Most sequences were estimated using direct sequencing, except in cases where individuals were heterozygous for an indel on each allele (or otherwise difficult sequences), where we used bacterial sub-cloning and sequenced 5–10 clones to estimate the correct genotypes. We estimated haplotype phase and identity among individuals using the recombination model of Phase [48] and a phase probability of 0.6.

Each of our 12 microsatellite loci had a di-nucleotide repeat motif. Tests of linkage between our microsatellite loci have been examined previously, and did not suggest restricted utility of these loci [49]; primer sequences and thermal cycle conditions are available there as well. PCR reactions for the microsatellite loci included 20 mM Tris–HCl (pH 8.4), 50 mM KCl, 1.5 mM MgCl_2_, 200 μM each dNTP, 0.25 μM each primer, 0.24 μL of 10 mg/mL bovine serum albumin (New England Biolabs), 0.5 U of taq polymerase (Biolase), and 1 μL DNA extract (10–50 ng⁄μL) in 6 μL reactions. Reactions were assembled in 384-well plates using the Matrix PlateMate 2 × 2 (Thermo Scientific) and amplified on an MJ Tetrad thermal cycler (MJ Research). Each 384-well plate had at least four positive and four negative control samples. One primer for each locus had one of four fluorescent dyes, and fragment sizes were determined in three runs per sample on an ABI 3730 Automated Sequencer (Applied Biosystems). Genotypes were scored using Genemapper (Applied Biosystems).

### Sequence analysis

**Figure 2:**
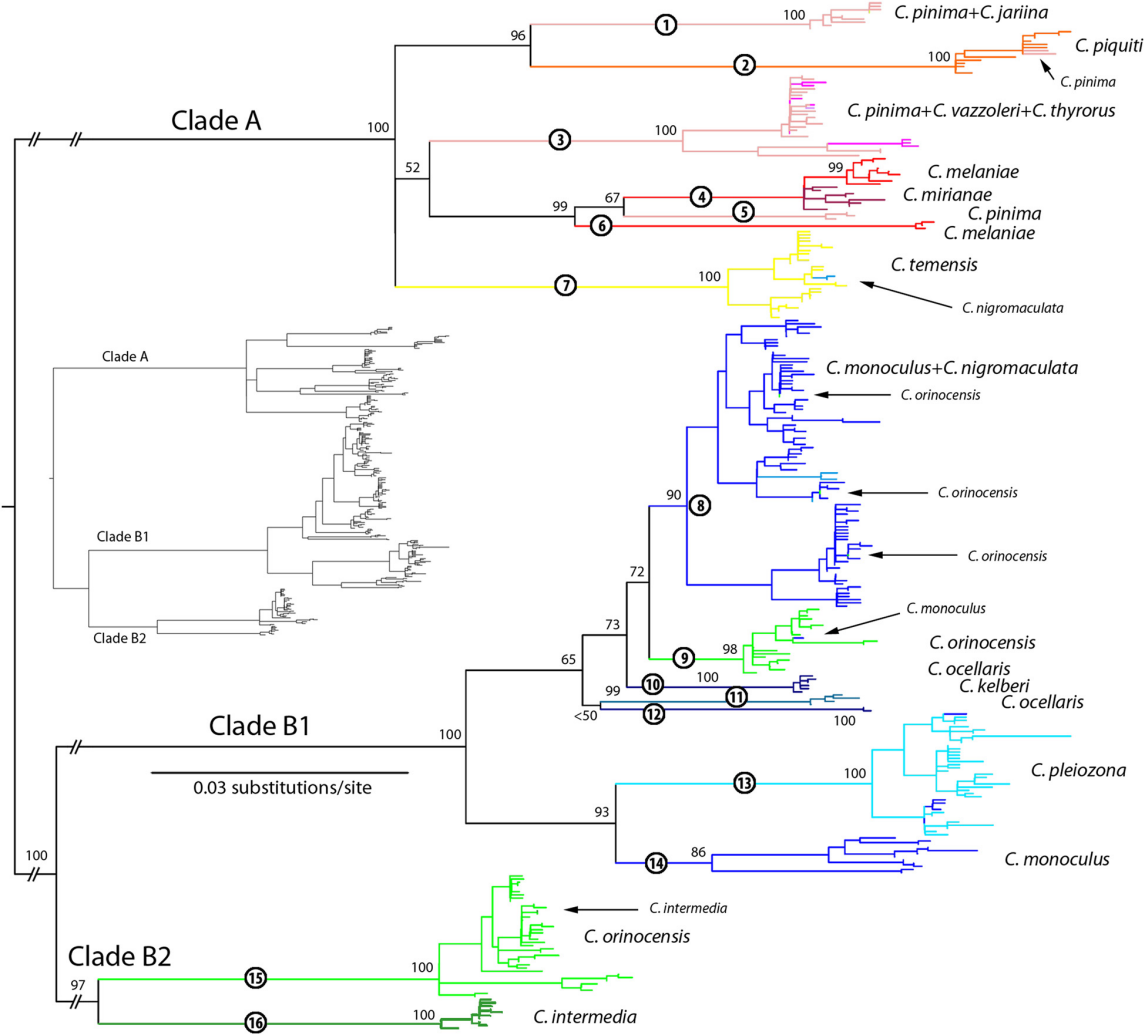
**Mitochondrial genealogy inferred from Treefinder, with haplotypes as terminals.** The tree is a maximum likelihood phylogram. Labeled branches (circled numbers) are referred to in the text. Stable, major mtDNA lineages are labeled (A, B1, B2). Branch colors correspond to the described species, and instances of morphotype-lineage mismatch interpreted as recent introgression are identified with arrows and smaller text. Branch values are bootstrap percentages. Note: basal branches of the tree are truncated for presentation, while the inset shows the non-truncated tree.

Based on previously published mtDNA genealogies [27,30] (also see Figure 2), mtCR sequences were divided *a priori* into 4 groups for alignment with the L-INS-i algorithm of MAFFT [50]: 1) *Cichla orinocensis* (clade B2), 2) *C. intermedia* (clade B2), 3) *C. ocellaris* + *C. monoculus* + *C. pleiozona* + *C. kelberi* + *C. nigromaculata* (clade B1), and 4) *C. temensis et al.* (clade A). We aligned these separately because although the L-INS-i algorithm is highly accurate, it has limitations for the number of sequences that can be aligned at one time. Product alignments were checked by eye to ensure the placement of gaps was consistent (isologous) among sequences. Using these four alignments separately, we identified unique sequences (haplotypes) using TCS 1.21 [51], treating gaps as a fifth state. Alignments of the unique sequences were combined together and aligned again using L-INS-i. Where available, the mtATP sequence of one individual bearing a haplotype was concatenated on to the mtCR sequence. The mtATP sequences did not vary in length and did not require additional alignment. We estimated appropriate models of sequence evolution for each of these two partitions (mtCR, mtATP) in Treefinder v. Jan2008 [52]. These were HKY + Γ and TN + Γ respectively. We then inferred a maximum likelihood phylogeny under these models in Treefinder using 1000 search replicates. We estimated support for major branches using 100 bootstrap replicates in Treefinder, but included only those sequences with both mitochondrial loci in this analysis; this nevertheless allowed us to estimate support for most clades of haplotypes. For the nuclear sequence loci, models of evolution and maximum likelihood genealogies were estimated for each locus using Treefinder as above (Mitf: HKY; Xsrc: HKY + Γ). We estimated branch support for these loci with 500 bootstrap replicates. Trees were mid-point rooted only for convenience of presentation (although this agreed with outgroup rooting for the mtDNA tree), and this rooting did not affect interpretation. Gene tree topologies were used to identify gene exchange (putative hybrids or intraspecific population admixture), detectable when localities and/or species designations are admixed across branches in the genealogy (i.e. multiple localities/species on a single branch, or a locality/species in more than one clade). Using these three genealogies, we looked for exclusive or private haplotypes or haplotype lineages that corresponded to putative species (i.e. monophyletic groups, or sets of polyphyletic clusters that were only exhibited by one geographically and/or morphologically circumscribed set of individuals). We also identified hybrids by mismatch between morphology (species ID) and genetic lineage.

### Microsatellite analyses

To further identify populations within which individuals exhibit co-ancestry and which localities are connected by gene flow, we analyzed the microsatellite genotypes using the Bayesian clustering program Structure [53]. This program attempts to match individuals to clusters that best correspond to a model of Hardy-Weinberg and linkage equilibrium, a model that implies a high degree of gene flow within clusters but lower or no gene flow between clusters. However, where admixture between clusters is indicated, this also represents co-ancestry between those individuals, and by extension, gene flow between the localities from which they were collected. Using this approach, introgressive hybridization could be detected when individual localities showed admixture between clusters that were otherwise distinct (i.e. non-overlapping sets of localities). Moreover, the two distinct clusters of individuals had to be sympatric or adjacent at these admixed localities to permit heterospecific mating. The program Structure has been extensively applied in tests of population structure e.g. [54], as well as species boundaries e.g. [55]. We divided the microsatellite data into clades A and B (sequence groups 4 versus 1–3 from above, respectively) to avoid the effects of fragment size homoplasy over larger phylogenetic distances [56]. With Structure, we made 20 runs of the program with each K (number of clusters) from 1 to 10. As most of these analytical constructions resulted in an asymptotic increase in the log probability of the data with increasing number of clusters (LnP(D|K)), we used the second order rate of change between runs of different K (ΔK) to estimate the optimal value of K [57]. We ran the program with the *r* (locality) prior implemented [58]. Posterior values of this prior between 0 and 1 indicate that locality data are informative for clustering, while values above 1 indicate that they are not. We made these runs with an initial value of *r* at 1 and an upper limit of 100. We found that it took much longer for this parameter to converge than is typical for other parameters in Structure (pers. obs.), so each run was made with 1 million sample generations after 1 million generations of burn-in. Evanno *et al.* (2005) found that their metric, ΔK, identified the optimal clusters at the highest hierarchical level in the data; inferring subsequent structure required dividing the original dataset. Thus after each series of runs, where ΔK indicated discrete clusters (clusters with a low degree of overlap, i.e. few individuals with split posterior assignment probability) we divided the data according to the clusters and made another series of runs as above. As this metric cannot indicate K = 1 as optimal, we continued to divide and reanalyze the data until the inferred optimal clusters showed a significant degree of admixture and geographic overlap, or LnP(D|K) showed a maxima for K less than K = 10. For each optimal K for each division, we averaged the posterior probability of individual assignment across all 20 runs using Clumpp [59].

We also analyzed each microsatellite dataset using another Bayesian clustering program, Structurama [60], following advice from a recent comparative review [61]. Structurama differs from Structure in that rather than requiring the user to specify *a priori* the number of clusters to which individuals should be assigned, Structurama uses a Dirichlet process prior for cluster assignment, allowing the number of clusters to be a random variable (albeit with a prior distribution) also sampled by the chain. As this program is quite similar to Structure, and our results were not significantly different, the analytical details and results of the Structurama analyses have been presented in the supplemental information (Additional file 4: File S1).

### Genomic extent of introgression

In cases of putative recent hybridization, we wanted to examine the degree of introgression of the nuclear genome. We again analyzed our microsatellite data using Structure, but in these analyses we used only data from the putative hybridizing localities and nearby non-hybridizing localities. Further, for individuals from non-hybridizing localities, we specified their species of origin and updated allele frequencies in the analyses only using these individuals. The analyses then estimated what proportion of the genome of the putative hybrids was derived from each of the two parental species. These analyses were run for 100,000 generations after 100,000 generations of burn-in, without the *r* prior. We made several runs to confirm posterior proportions across runs, but present the results of a single run. We tested whether or not cluster probability of hybrid individuals to one cluster or the other was significantly less than a mean expectation of 0.9 or 0.95 using one-sample *t*-tests.

## Results

### MtDNA genealogy

We sequenced the mtCR (aligned 563 bp) for 1,130 individuals of *Cichla*, including data from our previous publications. To this we added the 19 haplotypes from the 47 individuals of *C. monoculus* and *C. pleiozona* surveyed by Renno *et al.*[47]. Removal of redundant sequences from each of the four sequence sets aligned separately resulted in 11 haplotypes in *C. intermedia*, 61 haplotypes in *C. orinocensis*, 154 haplotypes in *C. monoculus* and the remaining clade B1 species, and 98 haplotypes from *C. temensis* and the remaining clade A species, for a total of 324 terminals. Overall, these haplotypes ranged from one mutation or gap to 16% uncorrected sequence divergence. Our search for the maximum likelihood genealogy, in which 121 terminals had mtCR and mtATP (842 bp) concatenated, resulted in a tree with LnL −9497.713 (Figure 2; Additional file 5: Figure S2a-c includes individually labeled terminals). Sequences used in this analysis are available from Genbank (DQ841819-DQ841946, GU295691-GU295801, JQ926745-JQ926928) and the tree and concatenated matrix are available from Treebase.org (#12624). The locations of terminals with both mtCR and mtATP sequences were well distributed across the tree. The mid-point root and topology of this tree agreed with our previous outgroup-rooted analyses that used an mtDNA dataset with fewer individuals, but included the cytochrome *b* gene, and partitioned the data by locus and codon [32]. As in our previous analyses [32,34], three major lineages (A, B1, and B2) are defined by the mtDNA data set but, with the new data, 16 divergent monophyletic groups of haplotypes are highly supported (Figure 2). These 16 clades are distinguished by the length of their subtending branches (number of mutations separating them from other such groups) and their restricted distribution geographically and/or morphologically (taxonomically). These mitochondrial DNA lineages are hereafter referred to as MTLs.

In this genealogy of mtDNA haplotypes, we discovered five distinct but not mutually exclusive patterns. The first pattern was that many putative species had exclusive or nearly exclusive (>95%) haplotype lineages that were a minimum of six (uncorrected) mutations different from any other species, and usually many more: *C. pinima* (Figure 2: MTL 1, 3, and 5), *C. piquiti* (MTL 2), *C. mirianae* (MTL 4)*, C. melaniae* (MTL 4 and 6)*, C. temensis* (MTL 7)*, C. ocellaris* (MTL 10 and 12)*, C. kelberi* (MTL 11)*, C. orinocensis* (MTL 9 and 15)*,* and *C. intermedia* (MTL 16). Haplotype lineages of these putative species were monophyletic, or paraphyletic with respect to haplotypes from other species, and some species (e.g. *C. orinocensis*) exhibited several polyphyletic haplotype lineages (see below). Due to the rapid coalescence rate of mtDNA, we interpreted this exclusivity and topology to imply a moderate degree of evolutionary independence among these putative species.

Second, several putative species exhibited haplotypes that were shared with, or one mutation different from, haplotypes of another species in the lineages identified above. These haplotypes made up the minority (<5%) of the total individuals of each species (identified in Figure 2). These were: *C. nigromaculata* (Mavaca, MV, 10 of 10 individuals) that exhibited *C. temensis* haplotypes (MTL 7); *C. intermedia* (Parguaza, PZ, 2 of 2) that exhibited *C. orinocensis* haplotypes (MTL 15); *C. pinima* (Tocantins, TO, 3 of 4) that exhibited *C. piquiti* haplotypes (MTL 2); *C. orinocensis* (Preta da Eva, PE, 6 of 6, and Novo Airão, NA, 3 of 11) that exhibited *C. monoculus* haplotypes (MTL 8), and *C. monoculus* (Novo Airão, NA, 1 of 11) that exhibited *C. orinocensis* haplotypes (MTL 9). Importantly, in all of these cases these individuals were sympatric with individuals of the species whose haplotypes they exhibited, or from localities adjacent to localities where the other species was found. Based on the rapid mutation rate at this locus, the geographic distribution of genetic overlap, and the otherwise high degree of exclusivity of haplotypes in these putative species, we inferred this haplotype sharing to be evidence of recent introgressive hybridization.

Third, several described species shared many or all of their haplotypes with other species, or had exclusive haplotypes that were one mutation away from, and nested among, the haplotypes of another species. These included: *C. nigromaculata* haplotypes that nested among haplotypes from *C. monoculus* (MTL 8); and haplotypes from *C. jariina*, *C. thyrorus*, and *C. vazzoleri* that nested among haplotypes of *C. pinima* (MTL 1 and 3). This pattern suggests that these species have exchanged genes so recently and to such a degree as to be indistinguishable.

Fourth, one pattern was exhibited only by *C. pleiozona* (MTL 13) and *C. monoculus* (MTL 8, 13, and 14). While a large portion of individuals classified as *C. monoculus* (240 of 324, 75%) exhibited haplotypes from a large monophyletic group (MTL 8), all but one fish from the middle Tapajós River (Jacareacanga, JC), middle and lower Madeira River (Aripuanã, AP, Humaita, HU, Cunia, CU, and Canumã, CM), and middle and upper Purus River (Boca do Acre, BA, Labrea, LB, and Tapauá, TP) exhibited haplotypes that were more closely-related to the haplotypes from nominal *C. pleiozona* (MTL 13 and 14). This included haplotypes that were only one mutation different between the two putative species (MTL 13). The remaining individual from the Canumã exhibited a haplotype from the main *C. monoculus* group (MTL 8), showing that these two groups of *C. monoculus* are not reproductively isolated (see also the microsatellite results, below). This pattern represents repeated, independent instances of unidirectional gene exchange between sub-populations in the upper Madeira and middle/lower Maderia and other adjacent Amazonas tributaries, likely following geodispersal of upper Madeira lineages facilitated by Andean orogeny, river channel meandering, and subsequent drainage capture. We interpret this to imply that *C. monoculus* and *C. pleiozona* exhibit historical and ongoing introgressive hybridization, or that they are sub-populations of a more inclusive species that intergrade at their boundaries, depending on the species concept employed.

Finally, the fifth pattern was four putative species that exhibited haplotype lineages that were exclusive to those species (except where described above), but polyphyletic. These were: 1) *C. orinocensis*, which exhibited two mtDNA clades, one sister to *C. intermedia* (MTL 15) and the other sister to the *C. monoculus* ‘main group’ (MTL 9). These two clades were parapatric (largely non-overlapping, but contiguous), with one northern clade found in the Orinoco and upper Negro, and the other southern clade found in the middle and lower Negro. Both clades were found together in the geographically intermediate Daraá (DA) locality; 2) *C. pinima (*sensu *lato)*, which exhibited 3 major clades: one sister to *C. piquiti* (MTL 1) that included *C. jariina*, another sister to *C. melaniae* + *C. mirianae* (MTL 3) that included *C. vazzoleri* + *C. thyrorus*, and a third nested among *C. melaniae* + *C. mirianae* (MTL 5). As in *C. orinocensis*, the two more common clades of *C. pinima* (MTL 1 and 3) were found sympatrically at two localities (Orixinimá, OX, and Tapajós mouth, TL), while the third clade (MTL 5) was restricted to a single locality (Vittoria do Xingu, VX), downstream from *C. melaniae*; 3) *C. melaniae*, with one more populous clade very closely related to the haplotypes of *C. mirianae* (MTL 4), and a second clade sister to the former clade of *C. melaniae* + *C. mirianae* + the Xingu clade of *C. pinima* (MTL 6). These two clades of *C. melaniae* were found sympatrically at both localities (Altamira, XA, and Iriri, IR) in the middle Xingu River drainage, more or less homogenously; and 4) *C. ocellaris*, which exhibited two clades of mtDNA, corresponding to the Essequibo and Maroni River (MA) drainages. In the first three cases (*C. orinocensisC. pinima s.l.*, and *C. melaniae*), the observations of significant genetic diversity within lineages and sympatry among lineages suggest that these clades result either from incomplete sorting of ancestral polymorphism (i.e. deep coalescence) or ancient introgression events [34], but in either case do not represent reproductive isolation among the two or more populations bearing those lineages because of the lack of strict geographical or morphological association. However, in the case of *C. ocellaris*, the geographic isolation of these lineages implies the presence of multiple evolutionarily significant units in watersheds of the Guyanas region.

### Nuclear gene trees

**Figure 3:**
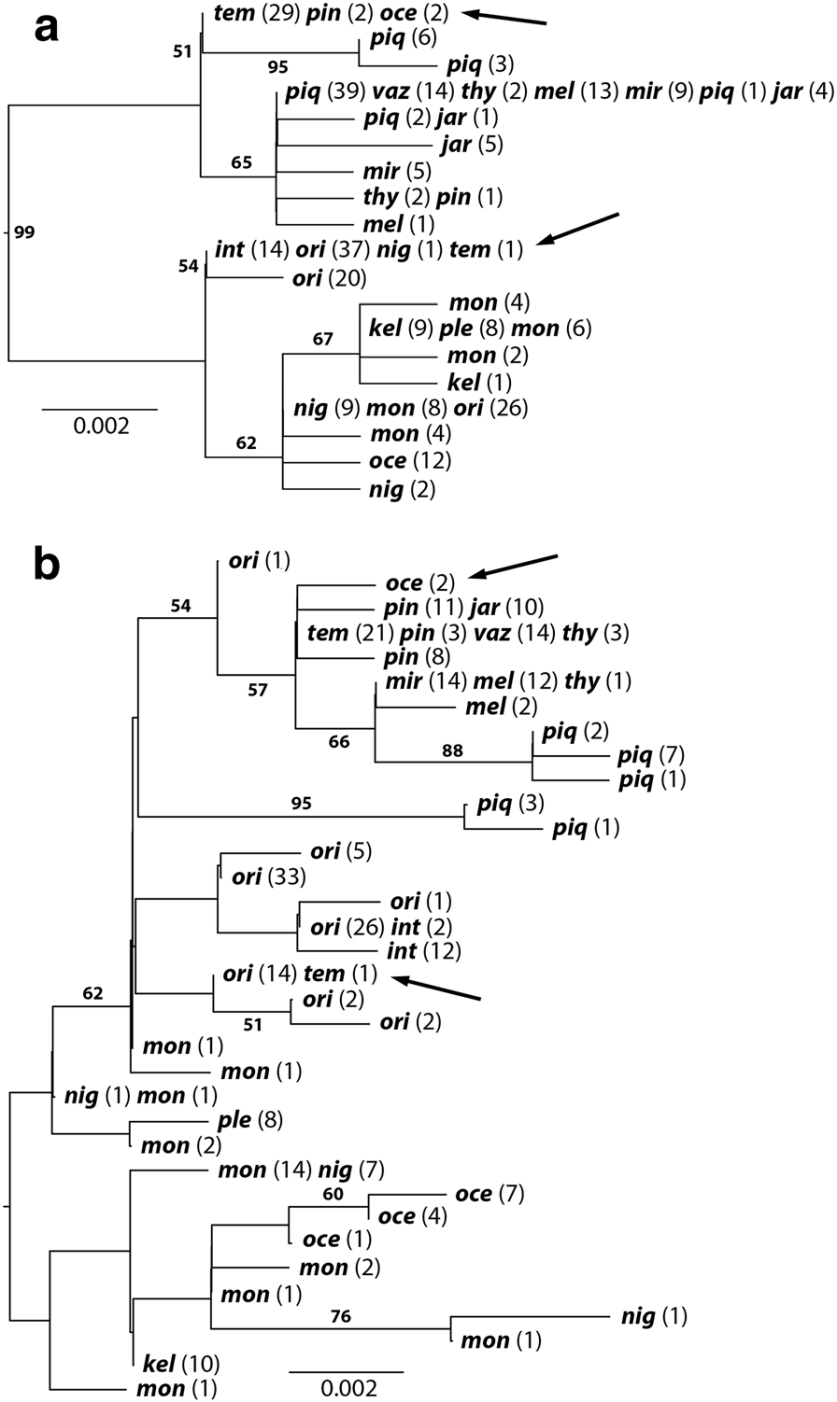
**Maximum likelihood genealogies for the nuclear genes. a**) Mitf and **b**) Xsrc. Terminals are haplotypes. For each haplotype, the number of alleles observed in each described species is listed. Branch values are bootstrap percentages.

We sequenced 150 individuals for the Mitf gene, which was variable at 24 sites along the 743 bp and resulted in 19 haplotypes (distinct alleles). Similarly, we sequenced 139 individuals for the Xsrc gene, which was variable at 32 of the 747 bp and exhibited 35 haplotypes. These are available from Genbank (JQ926929- JQ926982). Maximum likelihood genealogies of these loci were found with an LnL of −1196.07 and −1337.985 for Mitf and Xsrc respectively (Figure 3). In general, we observed few exclusive alleles or allele lineages among putative species, although alleles in the phylogeny did seem to mimic the major phylogenetic structure (Clade A, B1, B2). With so few mutations, it is difficult to distinguish the sharing of alleles within each clade as either gene flow or the incomplete sorting of ancestral polymorphism. However, these trees are useful for identifying introgressive hybridization between species in clades A and B. In both trees we observed that *C. ocellaris* from the Cuyuni River exhibited haplotypes more characteristic of *C. temensis*, while *C. temensis* from the Guri Reservoir on the Caroni River exhibited haplotypes characteristic of *C*. *orinocensis* (Figure 3). As above, these putative hybrids were either sympatric with individuals of the potential donor species, or adjacent to localities where they were found. However, these instances of hybridization were not detected by the mtDNA analysis.

### Microsatellite clustering

We genotyped a total of 1,034 individuals for the 12 microsatellite loci. These data are available from Dryad (http://dx.doi.org/10.5061/dryad.h4s73s5c). Individuals in this dataset had missing data at a maximum of 4 loci, resulting in 0.67% missing data in the overall dataset. In most cases, missing data corresponded to samples with dilute or partially degraded DNA. However, the exception was individuals from several localities for *C. pinima*, *C. vazzoleri*, and *C. jariina* that could not be amplified or scored consistently for locus CoriB6.2 despite repeated attempts. This may be indicative of null alleles at this locus, i.e. alleles that do not amplify due to mutations in the priming site. As the presence of null alleles in a heterozygous condition with amplifying alleles can bias estimates of heterozygosity and Hardy-Weinberg equilibrium, we repeated our analyses of clade A without this locus and observed qualitatively similar results. Otherwise, there was a significant variability at each locus in each clade of *Cichla* (Table 2), meaning these loci should be useful for estimating population connectivity. Several of the loci exhibited one base pair differences in fragment sizes, rather than the multiple of two base pair differences expected from di-nucleotide repeats. As these sizes persisted in samples that were re-amplified and genotyped two or more times, we scored alleles according to their electrophoresis mobility and made no attempt to force conformation to a two base pair sequence. We did not include samples from artificial reservoir habitats (nominal *C. temensis* from Guri Reservoir) in the following analyses.

**Table 2:** Allele diversity and size range for the microsatellite loci

	** *Clade A* **		** *Clade B1* **		** *Clade B2* **	
**Locus**	**alleles**	**size range**	**alleles**	**size range**	**alleles**	**size range**
Cint22	27	127-185	33	121-195	36	129-203
CoriA6	22	255-309	18	257-289	23	257-333
CoriB3	20	201-241	20	189-231	10	152-170
CoriB6.2	29	268-338	44	253-334	6	266-274
CoriD12	12	150-174	26	148-198	10	152-170
CoriF12	36	254-328	35	228-358	29	228-290
CoriG4	19	286-326	7	286-306	10	276-322
CpinC1	19	221-259	7	219-241	5	219-227
CpinC11	18	219-257	20	205-247	23	207-261
CpinD2	36	267-325	35	267-323	32	273-351
CpinE3	30	260-324	34	270-338	36	274-354
CichlaSM2	33	230-278	26	221-258	23	219-261

**Figure 4:**
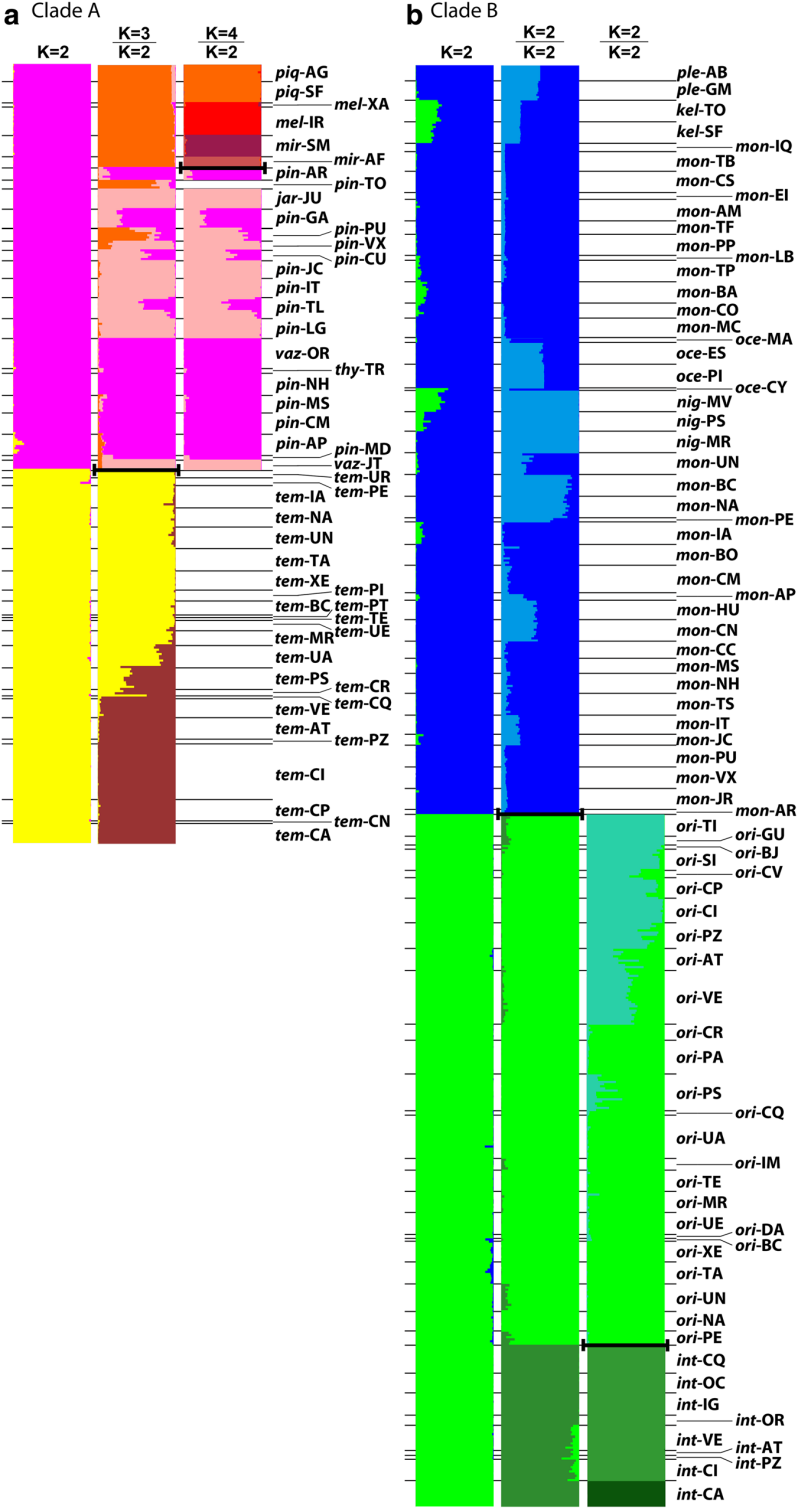
**Results of the microsatellite analyses, using the Structure divide-and-reanalyze approach.** Colors were chosen to represent the described species, but do not strictly correspond. Horizontal lines indicate the division of individuals from the same locality, which are identified by described species (first three letters of the species name) and locality abbreviation (Table 1). Bold lines indicate where data was divided for separate analysis, and K values above each column indicate optimal clustering on either side of the bold line. **a**) Clade A (N = 360). **b**) Clade B (N = 666).

Clade A: For clade A, that includes *C. temensis* and relatives (see Figure 2), we observed that the probability of the data given K in Structure (LnP(D|K)) continued to increase asymptotically as K rose from 1 to 10. Therefore, we determined the optimal number of clusters using the metric ΔK [57]. Graphs of LnP(D|K) and ΔK are presented in the supplemental figures (Additional file 6: Figure S3). The posterior value for *r*, the locality parameter, converged to less than one in every analysis, implying a significant degree of information content in the locality data. The optimal number of clusters for the entire clade A data, which included 360 individuals, was K = 2 (Figure 4a, first column). This resulted in two clusters with very little admixture that corresponded to 1) all nominal *C. temensis* (yellow) and 2) *C. pinima* plus the remaining clade A species (magenta). We divided these data and ran the program separately on each set. For *C. temensis* (Adiv1), ΔK indicated that K = 2 was optimal (Figure 4a, second column below heavy bar), but we saw a gradient in admixture from one cluster to the other (yellow and brown), indicating that a single cluster (population) was a better explanation of the data, albeit a population likely exhibiting isolation by distance from north to south along its extensive distribution (Additional file 1: Figure S1a). For *C. pinima* and the other species in clade A (Adiv2), ΔK indicated that K = 3 was optimal (Figure 4a, second column above heavy bar). Of these three clusters, two (Adiv2-1, pink, and Adiv2-2, magenta) included *C. pinimaC. jariinaC. thyrorus*, and *C. vazzoleri* with a significant degree of admixture between the two clusters, while the third cluster (Adiv2-3, orange) included *C. piquitiC. melaniae*, and *C. mirianae*. Two localities of nominal *C. pinima* exhibited a significant degree of assignment to this third cluster: Tocantins (*pin*-TO) and Paru (*pin*-PU) (note orange bars nested within the pink/magenta area, Figure 4a, second column). The Tocantins *C. pinima* were observed in the mtDNA tree to exhibit *C. piquiti* haplotypes (Figure 2, MTL 2), which suggests this microsatellite admixture also results from introgressive hybridization (*C. piquiti* are found adjacently, farther upstream in the Tocantins; Additional file 1: Figure S1a). For the Paru *C. pinima*, there was no evidence of haplotype sharing in the mtDNA tree. Further, these fishes are not adjacent to a locality where *C. mirianaeC. melaniae*, or *C. piquiti* are found, and intervening localities show no evidence of admixture. Looking at the data more closely, it appeared that the alleles that are exhibited in common between the Paru *C. pinima* and the Suia Missu *C. mirianae* (*mir*-SM, the most similar non-*C. pinima* locality) are also found in low to intermediate frequency throughout the distribution of nominal *C. pinima*. Thus it appears that these localities have independently evolved higher frequencies of these alleles, creating an artificial pattern of similarity.

To further understand structuring within these 3 clusters of Adiv2, we removed the samples of (hybrid) *C. pinima* from the Tocantins and analyzed separately the individuals from Adiv2-1 + Adiv2-2 (pink and magenta) and Adiv2-3 (orange). For this latter division, Adiv2-3, LnP(D|K) and ΔK both indicated that K = 4 was optimal, which corresponded to *C. melaniae* (red)*, C. piquiti* (orange)*,* and separated the localities for *C. mirianae* that lie in separate Amazonas tributaries (Suia Missu, *mir*-SM, and Alta Floresta, *mir*-AF) (Figure 4a, third column above heavy bar). There was also a smaller mode in ΔK at K = 2 that corresponded to *C. piquiti* vs. *C. melaniae* +*C. mirianae*. This indicates that while the *C. piquiti* localities are clearly connected by gene flow and separated from other populations, there is insufficient data (i.e. geographically intervening samples) to estimate connectivity between *C. melaniae* and *C. mirianae* in the Xingu River. For Adiv2-1 + Adiv2-2 (pink and magenta), ΔK indicated that K = 2 was optimal (Figure 4a, third column below heavy bar). We observed that the placement of localities in clusters did not correspond to geography in a simple way, or to current taxonomy. For example, the larger (magenta) cluster included most central localities, stretching from the Madeira tributaries (MD) to the mouth of the Amazon (AG) and, in addition to most *C. pinima*, also included nominal *C. vazzoleri* from Oriximiná (*vaz*-OR) and *C. thyrorus* from the Trombetas (*thy*-TR). The second cluster (pink) included the Tapajós localities (JC, IT) of *C. pinima* and those in or near the mouth of this river (TL, LG), but also, the non-adjacent *C. vazzoleri* from the Jatapu (*vaz*-JT) and *C. jariina* in the upper Jari (*jar*-JU). Other localities exhibited a split assignment to the two clusters, including the type locality for *C. pinima*, the Curuá-Una (*pin*-CU). This division into two overlapping clusters is congruent with the major pattern observed for these species in the mtDNA tree. This indicates that either these localities represent a single species with rather strong and complicated population structure or two species that are hybridizing in several localities.

Clade B: As above, the LnP(D|K) in Structure for the microsatellite data of clade B species, *C. ocellaris* + *C. orinocensis* and relatives, continued to increase asymptotically with K, so we used the rate of change between K (ΔK) to estimate optimal clustering. Also as above, the value for the locality parameter, *r*, was always estimated to be less than one. For the full dataset of 666 clade B individuals, the optimal K was K = 2 (Figure 4b, first column). This corresponded well with the mtDNA divisions B1 and B2, namely to *C. intermedia* + *C. orinocensis* (Bdiv1, green) and *C. ocellaris* + *C. monoculus* + *C. pleiozona* + *C. kelberi* + *C. nigromaculata* (Bdiv2, blue). There was some overlap between these two clusters at several localities of the *C. ocellaris et al.*, but most of these localities were not sympatric or adjacent to *C. orinocensis* or *C. intermedia*, implying it is probably a result of allele size homoplasy. After dividing the dataset for reanalysis, we found that the optimal clustering for Bdiv1 was K = 2, which corresponded to nominal *C. orinocensis* (light green) and *C. intermedia* (dark green) separately (Figure 4b, second column below heavy bar). Upon analyzing *C. orinocensis* separately (Bdiv1-1), K = 2 was determined to be the optimal clustering, but we observed a gradient in admixture moving from one end of this species’ distribution to the other (Figure 4b, third column above heavy bar, light green and blue green). As with *C. temensis* (Clade A), we interpret this to imply that a single cluster is truly optimal, possibly with isolation-by-distance along the species’ extensive distribution (see Additional file 1: Figure S1c). For *C. intermedia* (Bdiv1-2), we again found that K = 2 was optimal for these data (Figure 4b, third column below heavy bar), but in contrast to *C. orinocensis*, this clustering distinguished one sub-population of *C. intermedia* from the rest (*int*-CA). Upon examining the data from this locality, we observed in this locality’s data higher frequencies of alleles that were also present in lower frequencies at other localities, and a few unique alleles. As the mtDNA tree implied all of these individuals were very closely related, we did not further subdivide *C. intermedia* for analysis, considering it as potentially containing two or more evolutionary significant units (ESUs). For *C. ocellaris* and relatives (Bdiv2), we observed that the optimal number of clusters was K = 2 (Figure 4b, second column above heavy bar). This emphasized the distinctness of several localities in the Negro and Orinoco Rivers (all nominal *C. nigromaculata* and several *C. monoculus* localities) relative to the remainder (light blue vs. dark blue). However, a number of other localities also showed a significant degree of admixture between these clusters. We, therefore, did not divide and reanalyze these data.

### Microsatellite analysis of hybridization

**Figure 5:**
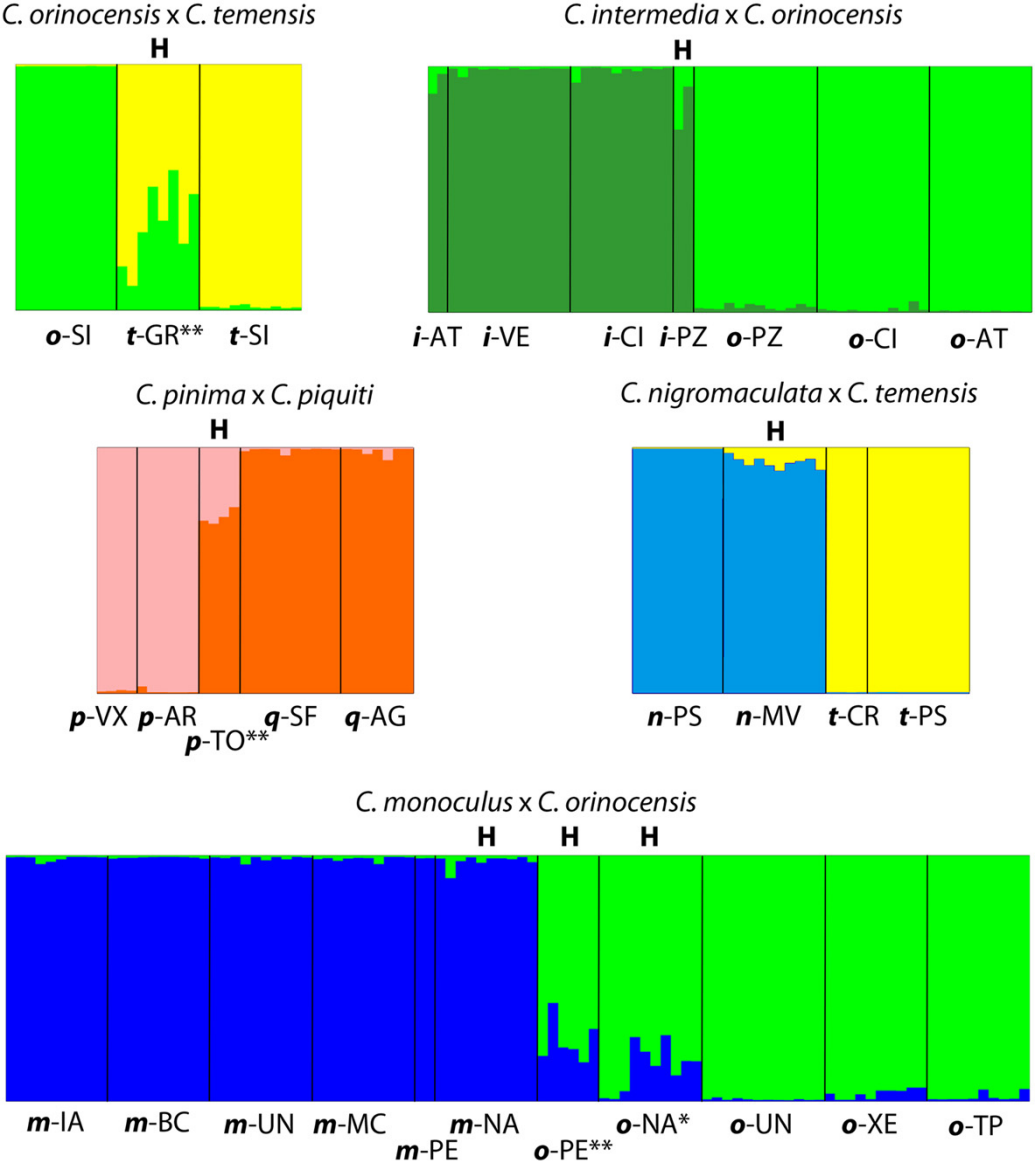
**Analysis of hybrid localities using Structure.** Allele frequencies were estimated only from non-hybrid individuals, and then used to estimate hybrid ancestry. ‘H’ above a locality denotes putative hybrid localities based on morphology, mtDNA, and/or nuclear sequences. * denotes mean assignment to parental species significantly less than 0.95; ** denotes significantly less than 0.9. Species and locality codes follow Table 1.

Based on the mismatch between morphology (species ID) and mtDNA or nuclear gene lineages, we identified 10 instances of gene exchange between described species, not including repetition at separate localities, involving nine described species (but see Discussion). These were *C. orinocensis*-*C. temensis*, *C. orinocensis*-*C. intermedia*, *C. orinocensis*-*C. monoculus*, *C. monoculus*-*C. pleiozona*, *C. ocellaris*-*C. temensis*, *C. nigromaculata*-*C. temensis*, and *C. pinima* with *C. piquiti*, *C. thyrorus*, *C. jariina*, and *C. vazzoleri*. We analyzed five of these putative hybrid sub-populations (those with sufficient sample size and that would not be affected by species delimitation) using Structure, along with the putatively non-hybrid individuals from adjacent localities (individuals from adjacent localities should have the most informative allele distributions for determining proportions of hybrid/non-hybrid ancestry of putative hybrids). These analyses showed a range of admixture (Figure 5). One-sample *t*-tests showed that some putative hybrids exhibited no significant nuclear admixture (cluster posterior > 0.95; e.g. *C. nigromaculata* at Mavaca, *n*-MV), while others exhibited introgression of nearly half their alleles (e.g. *C. temensis* in Guri Reservoir, *t*-GR). In the case of the nominal *C. pinima* from the Tocantins (*p*-TO), the nuclear genomes of hybrid fishes were actually more like *C. piquiti* than *C. pinima*, despite morphology! Some failed tests may have been affected by small sample size (e.g. *C. intermedia* at Parguaza, *i*-PZ). Others were significant only until additional non-hybrid localities were added (e.g. *C. orinocensis* at Parguaza, *o*-PZ), implying that population structure could also affect these tests.

## Discussion

### Species delimitation considering joint datasets

There is a growing consensus among evolutionary biologists and systematists that species should be treated as hypotheses that are subject to revision in light of data from natural populations [62,63]. Molecular data represent a useful resource in this context because they provide an assessment of effective genetic exchange between groups of individuals that are hypothesized to constitute an evolving biological entity [62]. Any set of data used to infer species boundaries, however, will suffer from the well-known taxonomic adage that the distinctiveness of two sets of individuals will often be the inverse of the number of specimens examined. In other words, apparent morphological or genetic discontinuities between small sets of specimens or distant localities are often sampling artifacts that may disappear as more specimens are examined. In effect, this implies that to adequately test species hypotheses, it is necessary to sample densely-enough, and adaptively, in a manner designed to test observed discontinuities between putative species [40]. In addition, the use of distinct data sources (morphology, mtDNA, microsatellites, etc.) provides a more robust test of species hypotheses since any one data source may provide a misleading estimate of cohesiveness or disjunction. Nevertheless, species, particularly widespread species, which often show significant geographically structured phenotypic and genetic variation, are contentious to delimit. Partly, this stems from the ambiguous correspondence between a species as a taxonomic category and the biological reality of populations of individuals [17]. Also, the reliance on static type materials to describe a dynamic, evolving population means that type series, and especially a single holotype, will only temporarily or perhaps never accurately capture the attributes of a species. Not surprisingly, this has lead to debates among systematists and other biologists as to how best to identify the contemporary slices of population lineages that we call species [13]. Yet, if we are to understand the role of hybridization and introgression in the evolution of biological diversity, we must be able to identify species and discriminate between intraspecific and interspecific mating and gene exchange.

In this case, we chose to compare and contrast results about species and introgression made under two different species concepts: the phylogenetic or diagnostic species concept (DSC) that recognizes species by the presence of diagnostic characters for morphological clusters of individuals, and a polytypic species concept (PTSC) that recognizes species as meta-populations (spatially bounded genetic clusters) and provides for the intergradation of ESUs that are adjacent. The PTSC is also largely consistent with the general lineage concept of species, which points out that as temporally horizontal cross-sections of meta-population lineages, not all sub-populations of a species should be expected to be exchanging genes at the present, though over the course of time various sub-populations may homogenize or go extinct without an overall change in alpha diversity [sensu 13, 38]. In an ideal case, where species are internally homogenous (panmictic and invariant) and externally discrete (diagnosable and non-admixing), various species concepts would identify the same units, but such cases are probably few and far between.

Using a DSC (called ‘phylogenetic’ in their manuscript, although without the use of true autapomorphies), *Cichla* was recently revised to include 15 species based on inferred morphological distinctness [25]. In contrast, using a PTSC, we interpret the combined data to support the discrimination of 8 species in the genus: *Cichla orinocensisC. intermediaC. ocellarisC. temensisC. melaniaeC. mirianaeC. piquiti*, and *C. pinima*. The remaining species appear to form species complexes within these taxa rather than independent biological entities (Table 3). We make the following provisional taxonomic recommendations, but recognize that they should be subject to further review of diagnostic characters. We consider the nominal species *C. monoculusC. pleiozonaC. nigromaculata*, and *C. kelberi* to be subspecies or evolutionarily significant units (ESUs) of *C. ocellaris* sensu *lato*. As *C. ocellaris* Schneider, 1801 was the first valid species of *Cichla*, this name would apply based on the rules of precedence. Similarly, we suggest that *C. jariinaC. vazzoleri*, and *C. thyrorus* are better considered synonymous designations of *C. pinima* sensu *lato*. In this case these taxa were all described in a single review, so the nomenclatural rules are ambiguous. However, as the nominal species *C. pinima* appears to show less incongruence given current results, we suggest that this name be used to refer to this species group.

**Table 3:** **Recommendations for a provisional revised alpha taxonomy of *Cichla***

**mtDNA clade**	**Described species**	**Recommendation**
A	*Cicha temensis*	consider valid
	*C. piquiti*	consider valid
	*C. melaniae*	consider valid
	*C. mirianae*	consider valid*
	*C. vazzoleri*	synonymize with *C. pinima*
	*C. thyrorus*	synonymize with *C. pinima*
	*C. jariina*	synonymize with *C. pinima*
B1	*C. ocellaris*	consider valid*
	*C. monoculus*	synonymize with C. ocellaris*
	*C. nigromaculata*	synonymize with C. ocellaris*
	*C. kelberi*	synonymize with C. ocellaris*
	*C. pleiozona*	synonymize with C. ocellaris*
B2	*C. orinocensis*	consider valid
	*C. intermedia*	consider valid*

Described species follow [25]. *May warrant further recognition of evolutionarily significant units.

Each of these delimited species is distinguishable based on morphology from the other delimited species (Figure 1b; see also [25]). Further, these species showed nearly exclusive lineages of mtDNA and separate clusters in the microsatellite analysis, implying that they experience, and have experienced in the past, more exclusive gene flow than with heterospecifics. For example, while *C. temensis* exhibited an optimal number of clusters of K = 2, many individuals were admixed between these clusters (Figure 4a). Further, this species showed mtDNA lineages that were exclusive to it (notwithstanding recent hybridization; Figure 2) and distributed heterogeneously throughout its range (Additional file 5: Figure S2a). Similarly, while *C. orinocensis* had two nearly exclusive clades (Figure 2), these were found together in one geographically intermediate locality (Additional file 5: Figure S2c), and there was no congruence with the transitions between mtDNA clades and microsatellite clusters geographically (Figure 4b). Moreover, for both *C. temensis* and *C. orinocensis*, within each of their mtDNA lineages, haplotypes were distinguished by many fewer mutations compared to hetero-specific haplotypes, suggesting a much more recent coalescence. As for the origin of the two mtDNA clades of *C. orinocensis*, we previously suggested these two be the result of an ancient introgression event [34], but incomplete lineage sorting cannot be dismissed outright. A multi-locus study is currently underway to distinguish these (Willis *et al.* unpublished data). In either case, the exclusivity of these clades (aside from limited introgression with *C. intermedia* and *C. monoculus*) and geographic overlap, coupled with the microsatellite results, implies a contemporarily separately evolving species.

*Cichla piquiti* and *C. melaniae* were each identified as single clusters in the microsatellite analyses, while *C. mirianae* was suggested to have two clusters corresponding to its sub-populations in the Xingu and Tapajós tributaries (Figure 4a). While *C. piquiti* had an mtDNA lineage that was well differentiated from other species, *C. melaniae* and *C. mirianae* had mtDNA haplotypes that were exclusive (i.e. no shared sequences) but paraphyletic and more similar than amongst other delimited species (≥6 mutations or ~1% sequence divergence at mtCR) (Figure 2). There was also some ambiguity in the microsatellite results as to whether these latter two species corresponded to one cluster rather than three (see graphs of LnP(D|K) and ΔK in the Appendix). Given our current sampling design versus the morphological disparity of these species (Figure 1b), we could not reject Kullander’s and Ferreira’s contention that there were two biological entities, but we recommend that a denser sampling in the middle and upper Xingu and upper Tapajós be done to further test the hypothesis that these are separate meta-populations. Similarly, two clusters were recovered for *C. intermedia*, with the second cluster including all samples from the Caura River (CA). We observed some unique microsatellite alleles for this population, including some loci that were fixed for a unique allele, but the mtDNA of these individuals show them to be relatively closely related to those from other localities (5–7 mutations or 0.9-1.2% sequence divergence from other conspecific haplotypes, versus 33+ uncorrected mutations (5.9%) from heterospecific haplotypes). This sub-population likely warrants conservation as an ESU, but the morphological consistency and minor genetic differences of these individuals suggests more evidence would be needed before rejecting the conspecificity of this sub-population.

The mtDNA of *C. pinima* was largely divided into two well-differentiated clades (Figure 2, MTL 1 and 3) that, while mostly allopatric, showed a complex pattern of geographical overlap and which were found sympatrically in two localities. The remaining clade of *C. pinima* mtDNA (MTL 5) was nested among divergent mtDNA lineages of *C. melaniae* and *C. mirianae* (MTL 4 and 6). Interestingly, this lineage was only collected from a single locality on the lower Xingu River (Vitória do Xingu, VX), downstream from where *C. melaniae* occur in the middle Xingu (XA). This restricted distribution, coupled with the presence of two divergent lineages in *C. melaniae* but not *C. mirianae* (in the upper Xingu, SM and upper Tapajós, AF), suggests that these lineages in both species (MTL 5 and 6) are remnants of an ancient introgression event. More data and further modeling will be required to confirm this, but in any event, the exclusivity of these clades (no haplotypes were actually shared by these species) and lack of significant admixture in their nuclear DNA (Figure 4a) suggests that they have experienced exclusive coalescence for a considerable period. The mtDNA of *C. vazzoleriC. thyrorus*, and *C. jariina* was subsumed within the two larger clades of *C. pinima* (MTL 1 and 3). Similarly, the microsatellite data for these four nominal species was best divided into two overlapping clusters (Figure 4a). While there was large congruence between these mtDNA and microsatellite datasets, it was not strict. For instance, while the nominal *C. vazzoleri* from the Jatapu (JT) were clustered with the southern *C. pinima* and *C. jariina* based on microsatellies (which show MTL 1), their mtDNA resided in MTL 3 with eastern *C. pinima*, Oriximiná (OX) *C. vazzoleri*, and *C. thyrorus* (Figure 2). The inference of two rather than four clusters and the complex geographical structure among them suggests that the described species have shared gene flow too recently to be evolving separately. Whether these two mtDNA and microsatellite clusters represent a single species with a very complex population structure (i.e. ancestral polymorphisms maintained by reduced gene flow among subpopulations) or two species with one or more zones of hybridization is unclear from the current data, but may be addressed through the use of coalescent-based models [64,65]. In any event, these species were originally distinguished on the basis of inconsistent differences in color pattern and overlapping meristic counts, and considering the present data, it seems more informative to consider them synonymous with a more inclusive species taxon (*C. pinima* sensu *lato*).

Similarly, for the species in clade B1, discrimination in the recent taxonomic work was based on very subtle difference in color pattern and overlapping meristic data. Here, we discovered that several of these putative species exhibited unique mtDNA lineages (Figure 2, MTL 10–12). Nevertheless, the microsatellite data did not distinguish these groups of sub-populations as being more dissimilar from each other (i.e. having a more exclusive history of gene flow) than some sets of sub-populations within nominal *C. monoculus* (Figure 4b). Moreover, there were several sets of sub-populations that showed incongruence between microsatellite affinity and mtDNA clade. For example, while several localities in the upper Purus River (BA, LB, and TP) and middle and lower Madeira River (CU, HU and CM) exhibited mtDNA more similar to the *C. pleiozona* clade (MTL 13 and 14), they always grouped with *C. monoculus* based on microsatellites (Figure 4b). Similarly, while middle Tapajós (Jacareacanga, JC) fishes had mtDNA more closely related to *C. pleiozona* (MTL 14), these fishes appeared more similar to *C. kelberi* with microsatellites (Figure 4b). It is important to note the split allegiance in molecular patterns could not be caused by misidentification due to ambiguous morphological description. In that case we would expect the molecular patterns to agree, albeit in conflict with the species ID. This suggests that despite having mtDNA lineages that coalesce rather deeply, there is little evidence for reproductive isolation between even the most divergent lineages within clade B1, since at every encounter following geodispersal, there is homogenization of the gene pools. While it is evident that some of these clade B1 sub-population groups show unique characteristics that imply a reduced rate of gene flow, several of the geographically-restricted nominal species (e.g. *C. ocellaris* from the Essequibo (ES) and Pirara (PI), *C. kelberi* from the Tocantins (TO) and São Felix do Araguaia (SF)) appear no more differentiated than sub-populations within more widespread nominal species (e.g. *C. monoculus* from Itaituba, IT) (Figure 4b). Rather, we interpret the data to imply that sub-populations in this clade form a widespread genetic mosaic. Under this interpretation, homogenous demes experience a gradient of gene flow with other such groups. At the high end, in this case closer to the main Amazonas channel, nuclear homogenization is common and mtDNA, while often developing unique haplotypes, is exchanged regularly among groups. At the low end, farther away from the Amazonas, gene flow is low enough (or isolation long enough) for divergence in mtDNA and moderate dissimilarity in microsatellite patterns. However, with the changes in river drainage through time (i.e. geodispersal), homogenization occurs between even more disparate sub-population groups. It is curious that these sub-populations on the fringe do not develop reproductive isolation, and this should be addressed with directed study. Perhaps the slow rate of molecular evolution in *Cichla *[66,67], or a tight correlation between morphology and a conserved niche, limits opportunities for divergence. In any event, the observation that these populations show few distinguishing morphological characters, appear ecologically interchangeable (e.g. occupy the same lagoon margin mesohabitat; SCW, personal observation) and are freely interfertile suggests that they should be considered subspecies or ESUs rather than separate species.

Our results differed from the morphological review of Kullander and Ferreira [25], a result we attribute not only to the consideration of molecular data, but also to the use of a different species concept and survey of an overall larger number of specimens. It is also important to point out that in the course of this study we had the advantage of reviewing the results of Kullander’s and Ferreira’s analysis and collecting data with an intent to test their hypotheses. On the other hand, there are a number of potential reasons why our dataset could be incorrect regarding species boundaries. Our sample sizes for some species were rather low, particularly for *C. thyrorus* (N = 2) and *C. jariina* (N = 9), each from a single locality. However, while the Structure analyses might be misled by this effect, low sample size would not explain the sharing or placement of mtDNA haplotypes from these individuals. Another reason for incongruence with current taxonomy could be the mutational constraint or bias of unknown strength that microsatellite loci are suspected to exhibit [68,69]. However, while there may be unknown biases in some populations, our observation of a significant degree of size variation and number of alleles in each species group implies that there should be no lack of power with these loci (Table 2). Moreover, while any one locus can provide a misleading estimate of population structure, our use of multiple loci and different analytical methods allowed us to estimate species limits while taking into account the idiosyncrasies of each data set.

### Discrimination and interpretation of introgressive hybridization in *cichla*

There is growing recognition that introgressive hybridization is a natural feature of many species, and that species can remain distinct over time despite chronic exchange of genes with other species [e.g. 12]. However, what largely remain to be elucidated are the overall rates of introgression among species and the near and long-term fates of heterospecific alleles. Answering this question requires large-scale genetic surveys and analyses that provide for genetic diversity to be interpreted in a historical context. The present data provide such an opportunity. Here, we consider the patterns of introgressive hybridization in *Cichla* under two different species concepts.

Based largely on the mismatch of morphology (species ID), mtDNA, and microsatellites, and to a smaller extent on nuclear gene genealogies, we identified 11 putative instances of recent introgression between species identified using the DSC (i.e. the species as recently revised [25]). Using mtDNA as a proxy, recent hybrid ancestry was evident for a small but significant proportion of the overall number of individuals in our dataset (139 out of 1,177 individuals showed morphology/mtDNA mismatch, ~12%, without considering ancient hybrids). This introgression involved 12 of the 15 described species of *Cichla* (80%). Based on relationships estimated from the mtDNA (Figure 2) and supplemented by phylogenies derived from nuclear DNA (Willis *et al.* unpublished data), introgressive hybridization shows a wide phylogenetic breadth, including both sister species (e.g. *C. pinima* x *C. jariinaC. monoculus* x *C. nigromaculata*) and more distantly related species (*C. temensis* x *C. orinocensis* or *C. ocellaris s.l*.).

When introgression is examined under the PTSC, some observations change, while others remain consistent. For example, under the PTSC, only 26 out of 1,177 individuals (~2%) exhibit heterospecific mtDNA; the others are considered to show conspecific mtDNA. On the other hand, while fewer individuals are of hybrid ancestry under this concept, a similar proportion of species experience introgressive hybridization, with 6 out of 8 delimited species acting as either the donor or recipient of introgression (not including putative ancient hybridization, which would be 7 of 8). Another consistent result was the phylogenetic breadth of introgression, which still included both sympatric and parapatric sister species (e.g. *C. orinocensis* x *C. intermedia*, *C. pinima s.l.* x *C. piquiti*, respectively) and species from different mtDNA clades. Thus it appears that despite the application of different species concepts, introgressive hybridization remains a widespread phenomenon, with only its overall numerical rate subject to change. In addition, the observation of apparent viability of hybrids between even more divergent species suggests that reproductive isolating mechanisms in this group, where they exist, are likely to be pre-zygotic.

As most hybrids were identified here using mtDNA, we were concerned that mtDNA would provide a biased estimate of the extent of introgression in *Cichla*. It has been suggested that mtDNA may introgress more readily than nuclear genes [6], perhaps inflating the apparent impacts of hybridization, although there are clear instances of the opposite phenomenon e.g. [70]. Where sample size permitted, the focused microsatellite analyses using Structure showed that introgression in the nuclear genome ranged from negligible to extensive (Figure 5), a pattern which did not seem to show a strong correlation with the degree of mtDNA introgression. This suggests that the forces governing introgression may be different in each case and depend on local circumstance (e.g. selection against hybrids). However, even if the only lasting indicator of introgression is mtDNA, this nevertheless shows that 1) early hybrids were viable and fertile and 2) subsequent backcrossing occurred. These points imply that opportunities for “adaptive introgression,” the transfer of adaptive mutations and an increase in genetic diversity not constrained by in situ mutation [71-73], are more widespread than is traditionally assumed. Particularly in species that are constrained by a slow rate of molecular evolution, such as appears to be true of *Cichla *[66,67], introgression may increase the genetic diversity and adaptive potential of a species and even stimulate lineage diversification [3,71,74-76].

There has been a recent campaign in the literature on species delimitation for species to be recognized based on quantitative disjunctions along multiple continuous axes of variation, and for all lines of evidence to be treated as equally (or potentially) valid indicators of separate evolutionary units. This stems from a desire by systematists to reconcile data emanating from population genetics, phylogenetics, ecology, morphology, and other disciplines, a recognition that species lineages are emergent properties of populations, and an acknowledgement that lineages are acted on by a heterogeneous array of evolutionary processes that logically produce a variety of outcomes [10,13]. The proponents of this paradigm recommend that species can be recognized by even one line of evidence, and that congruence of all indicators of species cannot be expected *a priori *[39]. However, as Padial and de la Riva point out, because various patterns mimicking species boundaries can arise as artifacts from processes unrelated to lineage divergence, “taxonomists need to be careful and critical with the evidence at hand…no one should consider a separate species to be a set of organisms that share a mutation in a gene, differ in morphology, or even appear to be reciprocally monophyletic, if there is evidence that those individuals belong to the same population or meta-population as others not showing such characters.” Moreover, in order to be consistent with the scientific method, it is not enough to be able to determine that two groups of individuals are different species; we must also be able to show that they are the same species. That is, species hypotheses must be *disprovable*. Unfortunately, it remains unclear what evidence should be considered sufficient for rejecting species, particularly when there is incongruence among datasets, since a crux of this philosophy (integrative taxonomy) is to avoid specifying the supremacy of certain data types. In fact there are many lines of evidence that may imply the sharing of a common gene pool between populations, such as incomplete reproductive isolation, admixture in genetic clusters, or non-exclusive coalescence of alleles, but without acknowledging chronic introgression as a process in many ways different from intraspecific gene flow, as others have pointed out, this would result in the sinking of many species generally regarded as otherwise ‘good’ biological units [e.g. 11]. Other researchers have recommended simply avoiding the whole issue of ‘species,’ and rather describing groups based on the amount they differ from other groups (e.g. mtDNA sequence divergence) [77]. While students of speciation might find this appealing, it is unlikely that this practice would ever catch on in the wider realm of biology and beyond. Thus, we are left with a question: if we are to continue under the philosophy that species are real and discoverable (even if they can’t all be expected to satisfy all possible criteria), are there natural distinctions between intraspecific gene flow and introgressive hybridization that can guide our delimitation of species?

The answer to this question, at least for *Cichla*, is a qualified ‘yes.’ When considering the borders of shared gene pools, as we did with the molecular data, introgression usually appeared as a distinct digression from patterns of exclusive co-ancestry, whereas gene exchange among conspecifics appeared as homogenous genetic clusters or broad regions of admixture between subpopulations. For example, consider several putative parapatric species from clade A: *C. pinima*, *C. jariina*, and *C. piquiti*. *Cichla jariina* showed no mtDNA haplotypes exclusive of *C. pinima* (Figure 2, MTL 1), and showed no significant deviation in allele frequencies from the overall microsatellite pattern of *C. pinima* (Figure 4a, column 2). In fact, the genetic structuring within *C. pinima* was much greater that the differentiation between *C. jariina* and nearby localities of *C. pinima*. In contrast, *C. pinima* and *C. piquiti* each showed haplotypes that were separated by long branches in the mtDNA tree (Figure 2, MTL 1 and 3 vs. 2), and their microsatellite clusters were clearly separated in the second step of the Structure analyses (Figure 4, column 2), as well as by Structurama (Additional file 7: Figure S4, Column 1). Their hybrids, on the other hand, were represented by a clear pattern of admixture in their microsatellites, and appeared as obviously mismatched individuals in the mtDNA tree. It should not come as a surprise, then, that *C. pinima* and *C. piquiti* adults are easily distinguishable based on morphology, while *C. pinima* and *C. jariina* are not (SCW, personal observation; see also Figure 1). Similar patterns of disjunction between intraspecific gene flow and introgression were seen in *C. orinocensis* and *C. intermedia* (also sister species). The qualification mentioned above applies to parapatric varieties such as *C. monoculus* and *C. pleiozona* or *C. nigromaculata*, and is that delimiters of species must keep an open mind when considering the scale of gene flow. While there may not appear a clear disjunction between types of gene exchange among the putative species in clade B1, when we recognize that the scale of meta-population dynamics (i.e. gene exchange, extirpation, colonization, and homogenization) likely includes all of the described clade B1 species, then the disjunction becomes more apparent. At this scale, as above, introgression between this (*C. ocellaris* sensu *lato*) and other delimited species appeared clear (Figures 2 and 4), while the boundaries between these sub-populations generally did not (Figure 4). However, we do not mean to suggest that some of these sub-populations (e.g. *C. kelberi*, or the Jatapu River population of *C. vazzoleri*) are not evolutionarily unique or are not in need of conservation as ESUs, as we are sure that they are. Rather, we infer that these units have not yet evolved (speciated) to the point where they are unique on the same scale as other species in the genus.

We also hope that our observations and conjectures stimulate investigation as to what conditions lead to the break down of reproductive isolation among *Cichla* species, and what are the fates of hybridizing populations. Considering the divergence in color pattern between our delimited species, this, perhaps coupled with some behavioral cues, seems a likely method of mate choice for these visual predators, but also one easily disrupted by transient changes in the environment e.g. [78]. In particular, several of our inferred instances of hybridization were from habitats altered by human influence. The *C. temensis* x *C. orinocensis* from Guri Reservoir (GR), for instance, experience genuine lacustrine conditions, a rare challenge for Neotropical fishes. Similarly, *Cichla pinima* x *C. piquiti* from the Tocantins (TO) were collected downstream of the Tucuruí Reservoir, a region which has experienced a radically different flow regime since the erection of the dam (based on conversations with local residents). The Cuyuni River (CY), where we inferred hybridization between *C. ocellaris* and *C. temensis*, has witnessed greatly increased sediment loads and reduced transparency due to dredging for gold (Willis, pers. obs.). Notably, for the Guri and Cuyuni fishes, our observation of morphology indicated that something was peculiar about these fishes, while all other inferred hybrids conformed to the morphology of non-hybrid parental individuals, again suggesting a role for selection in the fate of hybrid individuals. It is also worth pointing out that our method of detecting hybridization using molecular markers works well when the signature of hybridization is sustained for multiple generations, such as in a hybrid zone or where backcrossing has occurred (i.e. hybrid swarm or introgression), but is not effective for detecting transient hybridization where hybrids are inviable or infertile. As a result, hybridization in *Cichla* could be much more common than we realize. Nevertheless, our observations also suggest that, as noted earlier, species can apparently persist for long periods without fusion despite chronic, locally high but globally low levels of gene exchange with other species.

## Conclusions

We applied a qualitative method to assess the congruence between markers for genetic exchange and disjunction between putative species groups in order to delimit species in this widespread and morphologically conservative genus. We evaluated our results under both more exclusive and inclusive species concepts to investigate the frequency and extent of introgression across a species group. Rather than stress the species units we have delimited *per se*, we emphasize the genetic overlap between the described or delimited species, and call attention to the evolutionary processes that overlap implies. We observed that introgressive hybridization is likely a widespread but ephemeral phenomenon for populations in species rich faunas like Neotropical freshwater fishes, although its role in the adaptation and/or diversification of these fishes and other tropical lineages remains to be fully explored.

Based on extensive sampling and multi-locus analyses under a more inclusive species concept, we inferred that *Cichla* contains fewer species that correspond clearly to biological entities than are currently recognized. While at least two of these species contain evolutionarily significant units that are in need of conservation, these populations did not appear to be distinct in their mtDNA, microsatellites, or both. While some Neotropical fish species groups exhibit a smaller or more fragmented geographic distribution than *Cichla*, and could thus be expected to show a higher degree of microendemism, many are widespread and may experience genetic exchange over evolutionary timeframes. We recommend that systematists focusing on widespread Neotropical fishes work to test apparent morphological or molecular disjunctions before erecting novel specific categories.

## Competing interests

The authors declare that they have no competing interests.

## Authors’ contributions

SCW collected and prepared samples, analyzed data, and drafted the manuscript; JM developed the microsatellite loci and assisted in the collection and analysis of the microsatellite data; IPF facilitated the collection of samples and provided intellectual support for data interpretation; GO provided facilities and funds for sample and data collection, advised in the interpretation of data and construction and revision of the manuscript. All authors read and approved the final manuscript.

## Supplementary Material

Additional file 1 **Figure S1.** Maps of approximate distributions of the 15 described species of *Cichla*. Sample locations are indicated. Click here for file

Additional file 2 **Table S1.** Approximate coordinates and Atlantic versant of the localities sampled by the authors. For all other sites, see Renno *et al.* (2006). Click here for file

Additional file 3 **Table S2.** Availability of voucher specimens for samples used in this study. Interested parties should contact the collections listed directly for lot numbers. MCNG: Museo de Ciencias Naturales de Guanare (Edo. Portuguesa, Venezuela), AUM: Auburn University Museum (Auburn, Alabama, USA), ROM: Royal Ontario Museum (Toronto, ON, Canada), INPA: Instituto Nacional de Pesquisas da Amazônia (Manaus, AM, Brazil), MPEG: Museu Paraense Emilio Goeldi (Belem, PA, Brazil), CPUFMT: Coleção de peixes da Universidade Federal do Mato Grosso (Cuiaba, MT, Brazil). Click here for file

Additional file 4 **File S1.** Methods, Results and Discussion on Structurama. Click here for file

Additional file 5 **Figure S2.** Mitochondrial genealogy with localities and species where the haplotypes were observed. Tree is a maximum likelihood phylogram, and terminals are haplotypes. Localities follow Table 1, branch values are bootstrap percentages, and terminals with asterisks (*) included both mtCR and mtATP (see text). a) Clade A. b) Clade B1. c) Clade B2. Click here for file

Additional file 6 **Figure S3.** Plots of LnP(D|K) (left) and ΔK (right) for the divide-and-reanalyze Strucutre analyses. Click here for file

Additional file 7 **Figure S4.** Structurama and Structure comparisons. Left column: Results of the Structurama runs (no admixture) where K (no. clusters) was the number of clusters with the highest posterior probability. In each case, figures show Clumpp summaries of multiple runs where individuals are assigned to only one cluster; split assignment for an individual represents assignment to different clusters between runs. Right column: Result from Structure (with admixture) using the K that was optimal for Structurama. Locality codes follow Table 1, and species are abbreviated to the first three letters of their specific epithet. Colors follow Figure 4. a) Clade A (N = 360). b) Clade B (N = 666). Click here for file
