# Supplementary figures and images for: Simultaneous delimitation of species and quantification of interspecific hybridization in Amazonian peacock cichlids (genus cichla) using multi-locus data
Source: BMC Evol Biol. 2012 Jun 22;12:96. doi: 10.1186/1471-2148-12-96 (PMC3563476; doi:10.1186/1471-2148-12-96)

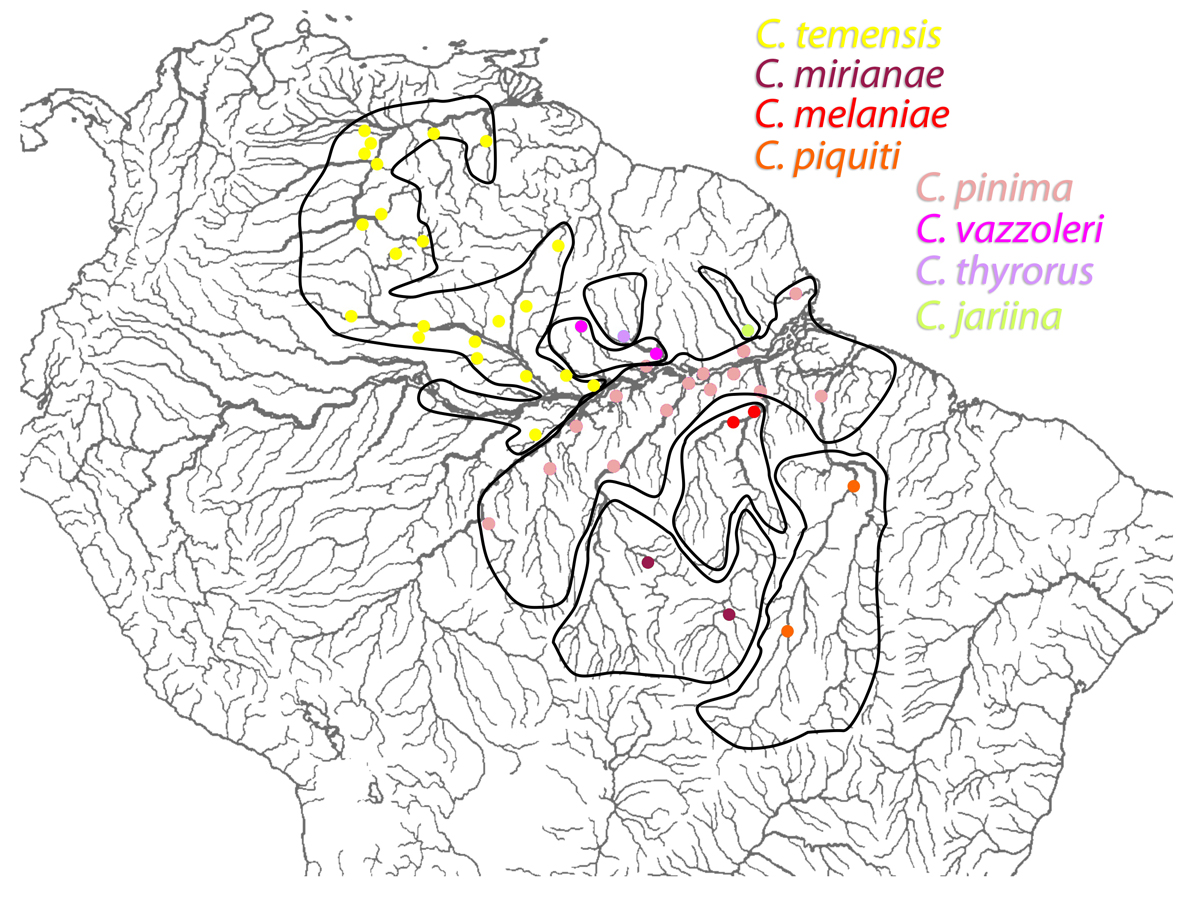

Supplement: Additional file 1 — Figure S1. Maps of approximate distributions of the 15 described species of Cichla. Sample locations are indicated. [file 1471-2148-12-96-S1.jpeg]

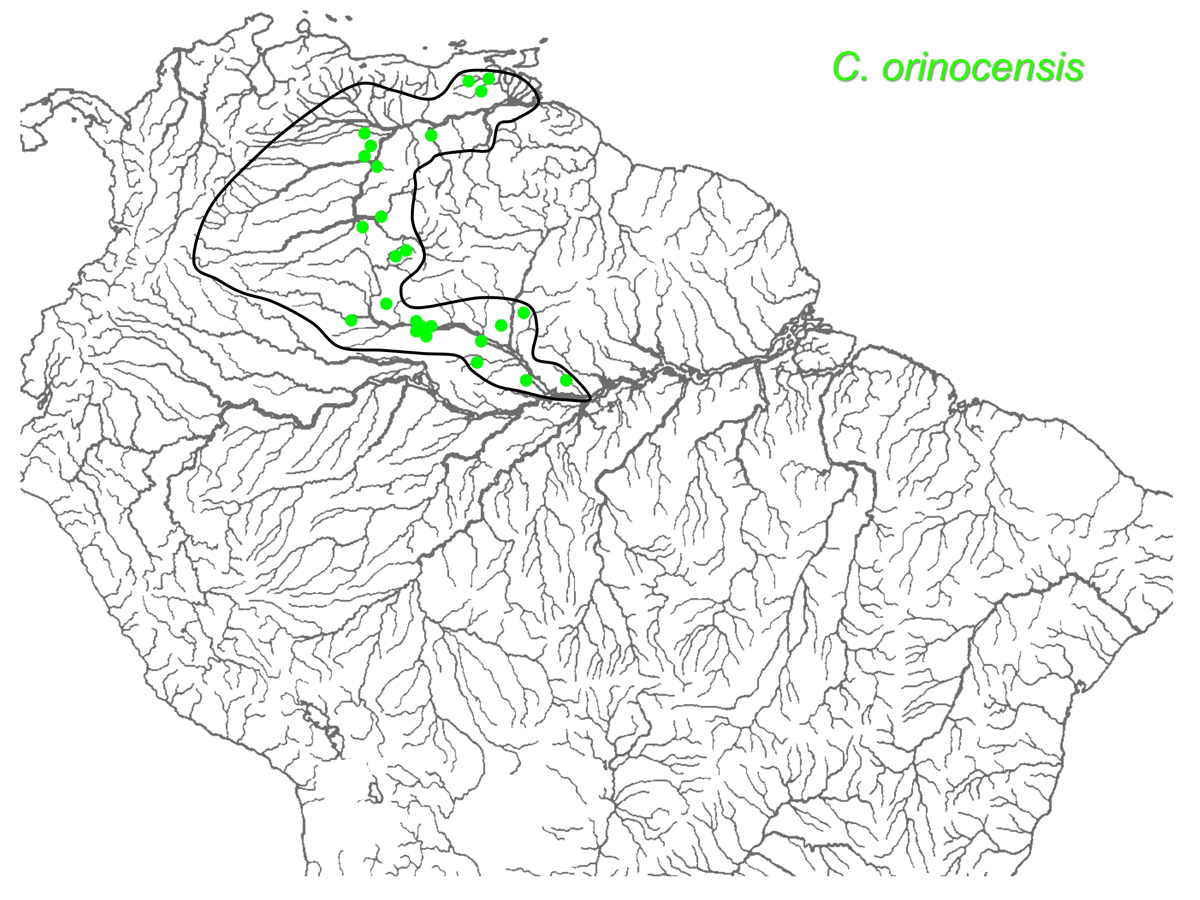

Supplement: Additional file 2 — Table S1. Approximate coordinates and Atlantic versant of the localities sampled by the authors. For all other sites, see Renno et al. (2006). [file 1471-2148-12-96-S2.jpeg]

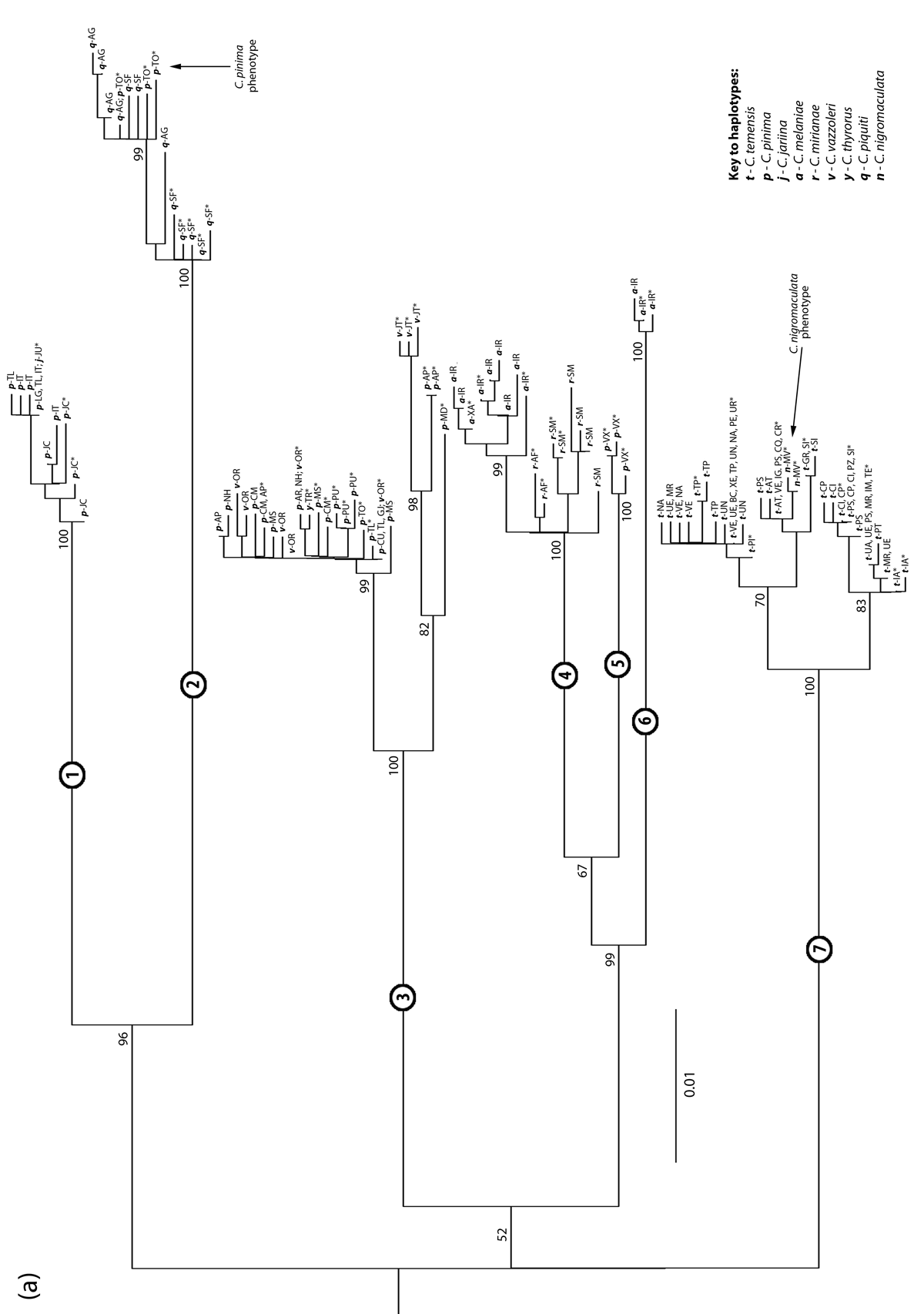

Supplement: Additional file 3 — Table S2. Availability of voucher specimens for samples used in this study. Interested parties should contact the collections listed directly for lot numbers. MCNG: Museo de Ciencias Naturales de Guanare (Edo. Portuguesa, Venezuela), AUM: Auburn University Museum (Auburn, Alabama, USA), ROM: Royal Ontario Museum (Toronto, ON, Canada), INPA: Instituto Nacional de Pesquisas da Amazônia (Manaus, AM, Brazil), MPEG: Museu Paraense Emilio Goeldi (Belem, PA, Brazil), CPUFMT: Coleção de peixes da Universidade Federal do Mato Grosso (Cuiaba, MT, Brazil). [file 1471-2148-12-96-S3.tiff]

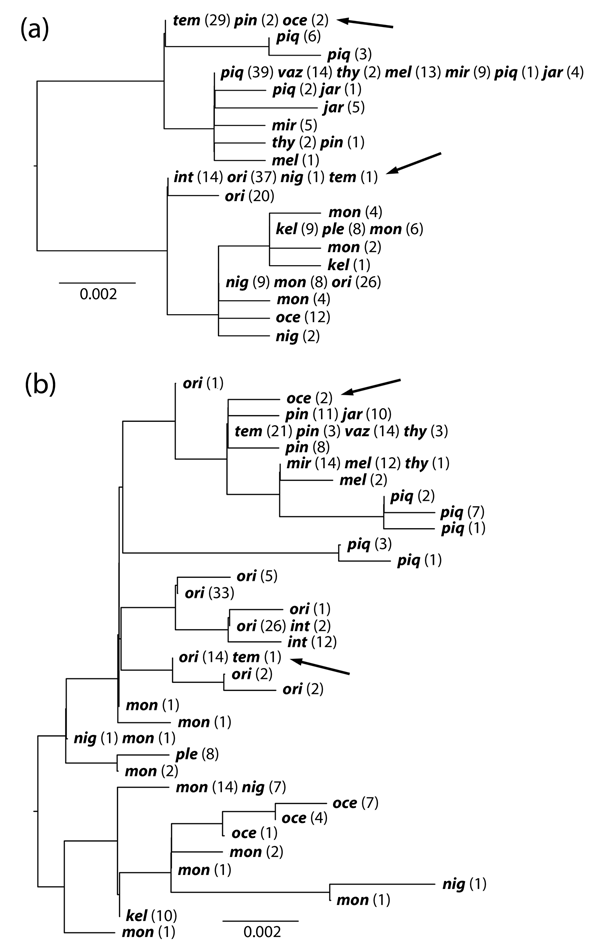

Supplement: Additional file 4 — File S1. Methods, Results and Discussion on Structurama. [file 1471-2148-12-96-S4.tiff]
