# Supplementary material for: Simultaneous delimitation of species and quantification of interspecific hybridization in Amazonian peacock cichlids (genus cichla) using multi-locus data
Source: BMC Evol Biol. 2012 Jun 22;12:96. doi: 10.1186/1471-2148-12-96 (PMC3563476; doi:10.1186/1471-2148-12-96)
Supplement: Additional file 7 — Figure S4. Structurama and Structure comparisons. Left column: Results of the Structurama runs (no admixture) where K (no. clusters) was the number of clusters with the highest posterior probability. In each case, figures show Clumpp summaries of multiple runs where individuals are assigned to only one cluster; split assignment for an individual represents assignment to different clusters between runs. Right column: Result from Structure (with admixture) using the K that was optimal for Structurama. Locality codes follow Table 1, and species are abbreviated to the first three letters of their specific epithet. Colors follow Figure 4. a) Clade A (N = 360). b) Clade B (N = 666). [file 1471-2148-12-96-S7.doc]

**Analysis of *Cichla* Microsatellite Data with Structurama**

**Analytical Construction**:

Structurama **(**Huelsenbeck & Andolfatto *Genetics* 2007, 175:1787-1802) differs from Structure (Pritchard, Stephens, & Donnelly *Genetics* 2000, 155:945-959) in that rather than requiring the user to specify *a priori* the number of clusters to which individuals should be assigned, Structurama uses a Dirichlet process prior for cluster assignment, allowing the number of clusters to be a random variable (albeit with a prior distribution) also sampled by the chain. With Structurama, we ran the two datasets (clade A and clade B) for 100,000 generations with 20 chains and a heating value of 0.03, sampling the cold chain every 100 generations and discarding the first 500 samples as burn-in. In initial tests, we found that runs of 1 million generations produced similar results. We ran each species group’s dataset treating number of clusters as a random variable, and made 4 runs each for prior means for number of clusters of 5, 10, and 15 expected clusters. We did not divide the data for subsequent runs as with Structure. We found it computationally intractable to run analyses with both admixture in individual assignment to cluster (where an individual can be assigned to more than one cluster, or in other words, its posterior probability split among several clusters) and number of clusters as random variables. However, the Structurama documentation suggests that when the admixture model is used with a fixed number of clusters, the model becomes equivalent to that implemented in Structure. We took the number of clusters with the highest cumulative posterior probability across all runs as optimal, and for those runs with this number in the mean partition, we summarized assignment of individuals to cluster across runs using Clumpp. To compare the variation in static (all or nothing) assignment of individuals to clusters using the optimal number of clusters with what would be expected under true admixture, we compared results from the Clumpp summarizations of the Structurama runs (with optimal K) to Structure runs with admixture for this same number of clusters (20 runs summarized using Clumpp).

Results:

Clade A:

Analysis of this clade A dataset with Structurama, without division, resulted in eight to nine clusters in the mean partition under a mean expectation of five clusters, nine to ten clusters under a mean expectation of ten clusters, and ten to eleven clusters under a mean expectation of fifteen clusters. As nine clusters was the most frequently observed number of clusters (highest cumulative posterior probability), we inferred this to be optimal for this dataset. However, assignment of individuals in those four runs that inferred nine clusters in the mean partition was not entirely consistent between runs. Therefore, the summary of assignment across runs was made with Clumpp (Supplemental Figure 4a), such that split posterior probability means assignment to different clusters across runs with the same overall number of clusters. Most of these clusters were similar to those found in the above divide-and-reanalyze Structure analyses. In addition, Structurama emphasized the distinctiveness of the Machado and Aripuanã *C. pinima* (*pin*-**MD** and *pin*-**AP**). However, as mentioned, this analysis did not allow admixture between the clusters; split assignment in this Clumpp summary represents assignment to different clusters between runs. Analysis of admixture in these data with K=9 using Structure showed a high degree of admixture between several clusters, in particular between Machado, Aripuanã, and other *C. pinima* localities (Supplemental Figure 4a).

Clade B:

Analysis of the clade B data with Structurama resulted in nine clusters with the highest cumulative posterior probability. Analysis with a mean expectation of five clusters resulted in seven to nine clusters with a posterior probability greater than zero, an expectation of ten clusters resulted in eight to twelve clusters, and an expectation of fifteen clusters resulted in nine to thirteen clusters. However, unlike in the analysis of the clade A taxa, analysis of the clade B data set did not support a single number of clusters in each individual run; rather, each separate run exhibited a divided posterior probability for number of clusters. For example, with an expectation of five, one run supported K=7:% 7, K=8: 37%, and K=9: 56%, with nine clusters in the mean partition. Summing the posterior probability across all twelve runs, nine clusters received the highest cumulative posterior probability, followed by twelve. For those five runs that exhibited nine clusters in the mean partition (highest posterior in that run), we summarized individual assignment to cluster across runs with Clumpp (Supplemental Figure 4b). As above, because this analysis did not allow admixture, split assignment in this Clumpp summary represents assignment to different clusters between runs even where there was the same number of clusters in the mean partition. In all Structurama runs, *C. orinocensis* and *C. intermedia* were separated from *C. ocellaris* and the other clade B1 taxa. In addition, this analysis emphasized the distinctiveness of several sets of localities in the *C. ocellaris* and relatives division (Bdiv2) in addition to those in the Negro and Orinoco: both localities of nominal *C. pleiozona* (*ple*-**GM** and *ple*-**AB**) plus two localities of *C. monoculus* in the middle Madeira (*mon*-**HU** and *mon*-**CU**); both localities of nominal *C. kelberi* (*kel*-**SF** and *kel*-**TO**); *C. ocellaris* from the Maroni (*oce*-**MA**); *C. ocellaris* from the Cuyuni (*oce*-**CU**); and *C. monoculus* from the Tapajós (*mon*-**IT** and *mon*-**JC**). However, several of these were assigned to different clusters between runs. Further, when these data were analyzed with Structure with K=9, some of the same localities remained distinct (e.g. *ple*-**GM**), while others were lumped together (e.g. *mon*-**IT**, **JC** with *kel*-**SF**, **TO**), and still others were emphasized as distinct but had not been before (e.g. *oce*-**ES**, **PI**). Moreover, there was a significant degree of admixture between these clusters. Taken together, these Structure and Structurama results portray localities of the *C. ocellaris* clade B1 (Bdiv2) along the main Amazonas as more homogenous (most nominal *C. monoculus*), while localities farther away from center, in the tributaries and satellite drainages, are more distinct (nominal *C. pleiozona*, *C. nigromaculata*, *C. ocellaris*, *C. kelberi*, and some *C. monoculus*). However, there was inconsistent clustering of localities into separate meta-populations (species), implying that one cluster is the overall best explanation for these five putative species.

**Discussion**:

We analyzed the microsatellite dataset by iteratively dividing the data and reanalyzing with Structure, and we found these results to be more directly informative than those from Structurama. In contrast, a recent review of multi-locus analyses of species boundaries using clustering approaches found that Structurama outperformed Structure when the optimal number of clusters were chosen in Structure using either LnP(D|K) or ∆K (Hausdorf & Hennig, Systematic Biology 2010, 59(5):491-503). This could suggest that our reliance on Structure in analyzing the microsatellite data was misguided. It was unclear from the cited study, however, how they constructed their analyses for each program and interpreted their results with respect to species hypotheses. We believe our divide-and-reanalyze protocol with Structure to be more consistent with the findings of Evanno et al. (Molecular Ecology 2005, 14:2611-2620), where ∆K was observed to identify the highest hierarchical level of structure in the data. Were one to interpret the first optimal clustering identified by ∆K as the best estimate of species, it is easy to see why this analysis could fail to identify species-level meta-populations. On the other hand, while the ability to estimate the number of clusters stochastically with Structurama is appealing, we found that analytical constructions without admixture may overestimate the number of clusters or provide ambiguous results. In the present case, with a dataset as large and complex as ours, we found it computationally intractable to implement the admixture model in our analyses with Structurama. However, when we examined the data in Structure using the number of clusters chosen using Structurama, we found that there was a high degree of admixture between many clusters, supporting the notion that using the admixture function may be beneficial for estimating the number of clusters in complex datasets.
